# Dysregulation of interaction between LOX^high^ fibroblast and smooth muscle cells contributes to the pathogenesis of aortic dissection

**DOI:** 10.7150/thno.66059

**Published:** 2022-01-01

**Authors:** Yinan Chen, Tao Zhang, Fang Yao, Xiang Gao, Dandan Li, Shufang Fu, Lin Mao, Fei Liu, Xuelin Zhang, Yongle Xu, Jianqing Deng, Weihao Li, Guangpu Fan, Cangsong Xiao, Yu Chen, Li Wang, Wei Guo, Bingying Zhou

**Affiliations:** 1 Shenzhen Key Laboratory of Cardiovascular Disease, Fuwai Hospital Chinese Academy of Medical Sciences, Shenzhen, 518057, China.; 2 State Key Laboratory of Cardiovascular Disease, Fuwai Hospital, National Center for Cardiovascular Diseases, Chinese Academy of Medical Sciences and Peking Union Medical College, Beijing, 100037, China.; 3 Key Laboratory of Pluripotent Stem Cells in Cardiac Repair and Regeneration, Chinese Academy of Medical Sciences, Beijing, 100037, China.; 4 Vascular Surgery Department, Peking University People's Hospital, Beijing, 100044, China.; 5 Department of Vascular Surgery, The Second Hosipital of HeBei Medical University, Shijiazhuang, 050000, China.; 6 Vascular Surgery Department, The first medical Center of PLA General Hospital, Beijing, 100853, China.; 7 Cardiac Surgery Department, Peking University People's Hospital, Beijing, 100044, China.; 8 Cardiovascular Surgery Department, The first medical Center of PLA General Hospital, Beijing, 100853, China.

**Keywords:** aortic dissection, single-cell RNA-sequencing, cell-cell interaction, fibroblasts, BMP signaling pathway

## Abstract

**Rationale:** While cell-cell interaction plays a critical role in physiology and disease, a comprehensive understanding of its dynamics in vascular homeostasis and diseases is yet absent.

**Methods:** Here, by use of single-cell RNA-sequencing and multi-color staining, we delineate the cellular composition and spatial characterization of human aorta with or without aortic dissection (AD).

**Results**: Scrutinization of cell subtype alterations revealed significantly changed fibroblast (FB)-smooth muscle cell (SMC) interactions in AD. Of these cellular interactions, LOX^high^ fibroblast (fibroblast subtype 2, FB2) in diseased state exerted the most pronounced effects on pathological deterioration of SMCs in AD. In addition, pharmacologically targeting the BMP (bone morphogenetic protein) signaling pathway effectively suppressed FB2 state transition and reduced AD incidence in mice. Finally, COL5A1 (collagen type V alpha 1 chain), one of the secreted proteins released from FB2, was significantly higher in the plasma of AD patients than in control patients, suggesting its potential use as a biomarker for AD diagnosis.

**Conclusions**: Our work not only identified a pivotal role of a specific FB subtype in AD progression, but also shed light on cell interaction dynamics in vascular diseases.

## Introduction

Aortic dissection (AD) is a life-threatening aortic disease associated with high morbidity and mortality. It is known that the aorta of AD patients undergoes pathological changes including loss of vascular smooth muscle cells, extracellular matrix (ECM) degradation in the tunica media, and inflammatory cell infiltration 1-4. Genetic disorders, such as Marfan syndrome, Loeys-Dietz syndrome and Ehler-Danlos syndrome, are important causes for AD 5. However, the molecular basis of non-inherited AD remains largely unknown.

Intimal tear marks the onset of acute AD, allowing blood flow into the media and induces oxidative stress due to hemoglobin and redox-active Fe^2+^ release 6. Abundant evidence suggests oxidative stress, as well as infiltration of immune cells and their secreted cytokines (e.g., interferon gamma (IFN-*γ*), interleukin-4 (IL-4), IL-9, IL-17, IL-22) 7, 8, be involved in vascular remodeling, and thus crucial mediators of AD. Both innate and adaptive immune cells, such as macrophages, neutrophils and lymphocytes, are known to promote the release of reactive oxygen species 9, 10. Oxidative stress in turn aggravates acute AD through causing chronic inflammation in the media by activating multiple transcription factors, including nuclear factor kappa B (NF-κB) 11-13, and is also known to participate in vascular smooth muscle cell phenotypic switching and apoptosis 14. Hypertension is another important risk factor of AD owing to surges in the mechanical stress of the arterial wall. Almost 80% AD patients have hypertension 15, 16. The ECM in tunica media sustains 95% pressure from blood flow 17. Abnormal smooth muscle cells (SMCs) synthesize and secrete abnormal ECM which is unable to sustain elevated blood pressure. However, the triggering event of SMC phenotypic switching and their subsequent loss is still unclear 2, 18-20. Therefore, the current status of AD treatment call continued efforts to scrutinize the molecular basis of AD pathogenesis.

Accumulating studies revealed critical roles of cellular heterogeneity in embryo development, cancer progression, stem cell fate decision, underscoring the importance to recognize cell diversity in physiology and disease at higher resolution 21-24. Recently, several studies classified macrophages, B cells and T cells into different subclusters with specific gene signatures which displayed distinct functions corresponding to different atherosclerosis stages 25, 26. In addition, cell atlases of the vasculatures in the mouse brain and lung were illustrated 27, 28. Thus, acquiring high-resolution snapshots of cell changes and molecular events in normal and diseased human arterial walls may hold the key to identifying pathways critically involved in AD 29.

To this end, we performed single-cell RNA sequencing (scRNA-seq) using human aorta with or without AD. We identified major cell subtypes and their distributions in the arterial wall, as well as their changes in AD. By analyzing intercellular communication, we unveiled pathways that are central to AD onset, and proposed the use of circulatory COL5A1 as a diagnostic biomarker for AD. This large-scale transcriptome dataset of human arterial cells is a useful resource for understanding the basic characteristics of AD, and for may be potentially exploited for AD diagnosis and intervention.

## Methods

All data, analytic methods, and study materials will be made available to other researchers for purposes of reproducing our results or replicating the procedures.

### Human samples

All human samples used for single-cell analysis were from the ascending aorta and included intima, inside dissected media, outside dissected media and adventitia. Non-AD aorta samples were collected from 3 male patients; one of them underwent heart transplantation, the other two underwent coronary artery bypass grafting. AD aorta samples were obtained from 4 Stanford A type aortic dissection patients (3 male, 1 female), underwent total arch replacement and stent implantation. The range of donor ages was 24-72 y, with a median age of 51 y.

Human blood samples were collected for enzyme-linked immunosorbent assay (ELISA). Blood samples from 30 healthy volunteers (23 male, 7 female) were analyzed as controls. Their ages ranged from 22 to 52 y, with a median age of 31 y. Eighty blood samples were collected from aortic dissection patients (66 male, 14 female; 6 Stanford type A, 74 Stanford type B); the age range was 26-83 y, with a median age of 52 y.

All the experiments were approved by the Ethics Committee of Peking University People's Hospital, as well as by the Ministry of Science and Technology. All patients received oral and written information about the study, and written informed consent was obtained before inclusion in the study.

### Cell isolation from human aorta

Samples were transported to the laboratory in ice-cold DMEM/F12 medium, then sliced into tissue sections (2 mm in length) and further triturated with sharp scissors into tiny fragments. The samples were digested with 2 mg/mL collagenase type I (Worthington-Biochem, LS004196) in a 37 °C water bath for 1.5 h. Digested cells were pelleted by gentle centrifugation, and the supernatant was discarded. Cell pellets were resuspended in 1 mL pre-warmed DMEM/F12 medium and processed for single-cell selection.

### Single-cell RNA-seq

Single-cell RNA-seq was performed as previously described 30. In brief, after staining with a mixture of Hoechst 33342 and Propidium Iodide (Thermo, R37610), cell viability was calculated; then, cells were centrifuged at 100 × *g* for 3 min at room temperature, and resuspended in an adequate volume of pre-warmed 1× DPBS to achieve the desired cell concentration. The cell dilution liquid (per 100 μL) was prepared using 2,200 cells, 1 μL of second diluent 2× (Takara, 430-000239-0001), 1 μL of RNase Inhibitor (Invitrogen, AM2694) and was diluted to a final volume of 100 μL with pre-warmed 1× DPBS. The diluted cells, together with other reagents, were dispensed into 384-well Nunc plates (Takara, 430-000025-20) for further analysis. Cell images were captured with Micro-Manager (Vale Lab), and were analyzed by utilizing CellSelect (Takara), and single, viable cells were selected for reverse transcription with 220 μL reaction mix prepared as follows: 56 μL 5 M betaine solution (Sigma-Aldrich, B0300-1VL), 24 μL 25 mM dNTP mix (Clontech, 639132), 3.2 μL 1 M MgCl_2_ (Thermo Fisher Scientific, AM9530G), 8.8 μL 100 mM dithiothreitol (SigmaAldrich, 43816-10ML), 61.9 μL SMARTScribe 5×First-Strand Buffer (Clontech, 639537), 33.3 μL SeqAmp 2× PCR Buffer (Clontech, 638504), 4.0 μL 100 μM RT E5 Oligo (Takara, 430-000240-0001), Amp Primer (Takara, 430-000241-0001), 1.6 μL Triton X-100 (Sigma-Aldrich, T8787), 28.8 μL SMARTScribe reverse transcriptase (Clontech, 639537) and 9.6 μL SeqAmp DNA Polymerase (Clontech, 638504). Then, 50 nL of this solution was dispensed automatically into each selected nanowell and synthesis of cDNA was carried out by using a Chip Thermal Cycler (BIO-RAD, 1861096) according to the manufacturer's instructions. The cDNA was further concentrated and purified using the DNA Clean & Concentrator kit (Zymo Research, D4004) and Agencourt AMPure XP magnetic beads (Beckman, A63880) sequentially. cDNA quality was assessed with Bioanalyzer (Agilent, 2100), and was quantified with the Qubit dsDNA HS Assay Kit (Invitrogen Q32851). As required, the cDNA product was diluted to about 0.5 ng/μL for library generation. Libraries were constructed using the Nextera XT Library Preparation Kit (Illumina, 15032350), and purified using Agencourt AMPure XP magnetic beads (Beckman, A63880). All of the libraries were sequenced using an Illumina NextSeq 500 sequencer.

### Immunohistochemical staining and immunofluorescence

Immunohistochemical and immunofluorescence staining was performed as previously described 31. Human and mouse aortic tissues were sliced into 3-μm-thick serial sections. Antibodies used were as follows: anti-LOX (abcam, ab31238) at 1:200, anti-PDPN (abcam, ab217886; Invitrogen, 53-5381) at 1:200, anti-THY1 (abcam, ab181469) at 1:200, anti-ADAM12 (Proteintech, 14139-1-AP-150) at 1:150, anti-ITGB1 (Proteintech, 12594-1-AP-150) at 1:150, anti-GPM6B (abcam, ab224318) at 1:100, anti-KCNMB1 (abcam, ab3587) at 1:150, anti- ITGA3 (abcam, ab134044) at 1:150, anti-VWF (abcam, ab6994) at 1:200, anti-PPIH (Proteintech, 11651-1-AP) at 1:150, anti-OC3 (abcam, ab181450) at 1:100, anti-F4/80 (abcam, ab6640) at 1:100, anti-COL5A1 (Proteintech, 67604-1-Ig) at 1:1000. At least three sections per aorta from three donors were used per staining. Alexa Flour 488 rabbit IgG and Alexa Fluor 594 - AffiniPure goat anti-mouse IgG secondary antibodies (Thermo Fisher Scientific) were used for immunofluorescence analysis. Immunohistochemical staining tissue sections were imaged by 3DHISTECH Pannoramic SCAN. Immunofluorescence sections were imaged using a Leica SP8 laser scanning confocal microscope. For multi-color immunofluorescence, Opal Multicolor IHC kits (PerkinElmer, USA) were used, following the manufacturer's instructions; and the stained sections were imaged by the Vectra Polaris system (PerkinElmer).

### Animal Experiments

All animal experiments were reviewed and approved by the Animal Care and Use Committee, Experimental Animal Center, Fuwai Hospital, National Center for Cardiovascular Diseases, China. Three-week-old male wild-type C57BL/6 mice were fed drinking water containing 0.5% β-aminopropionitrile monofumarate (BAPN) (Sigma-Aldrich, A3134) for 28 days followed by infusion with angiotensin II (AngII, 1000 ng/kg/min) by Alzet osmotic pumps (Alzet micro-osmotic pump, 1007D) for 48h. In this experiment, mice fed with water were used as controls. To perform intervention experiment, PDTC (Selleck, S3633), GW7647 (MCE, HY-13861), and Oroxin B (Selleck, S9190) was first dissolved in DMSO, at concentrations of 32 mg/mL, 60 mg/mL and 100 mg/mL, respectively. LDN-193189 (Selleck, S7507) was first dissolved in water at a concentration of 88 mg/mL. Then, these compounds were prepared by diluting in 20% Pluronic F127 (Sigma-Aldrich, P2443) saline solution with agitation at 4 °C overnight. Mice were randomized to receive different compounds. At the second week of BAPN administration, saline, 20 μL PDTC (50mg/kg), LDN-193189 (10 mg/kg), GW7647 (10 mg/kg) or Oroxin B (5 mg/kg) was added in a gel form on the aortic arch of each mouse during open chest surgery. In this experiment, mice treated with saline were used as control. After open chest surgery, administration of BAPN was continued. Infusion with AngII (1000 ng/kg/min) by Alzet osmotic pumps (Alzet micro-osmotic pump, 1007D) for 48h was followed after 4-week of BAPN administration. The maximal thoracic aorta diameter was measured with the VisualSonics Vevo 2100 imaging system. Blood pressure was monitored with a noninvasive tail cuff MRBP System (IITC Life Science, USA). All data were analyzed by two independent investigators blinded to the experiment.

Before pump implantation and open chest surgery, mice were anaesthetized with 2% 2,2,2-tribromoethanol (~350 mg/kg) with intraperitoncal injection. The adequacy of the anaesthesia was monitored by keeping track of the breathing frequency and the response to toe pinching of the mice. After pump implantation and open chest surgery, 5% lidocaine cream was given on the skin to relieve pain. Mice were sacrificed after 48h of AngII infusion by exsanguination under anaesthesia.

### Flow cytometry

Two human Stanford type A aortic dissection specimens were used for isolation of dFB2 to collect conditioned medium. Neither had connective tissue diseases such as Marfan syndrome, Ehles-Danlos syndrome, and aortitis, according to their clinical history and physical examination. Single cell suspensions of human AD adventitia were obtained by digestion with 2 mg/mL collagenase type I in a 37°C water bath for 1.5 h. To harvest FB2 cells from AD tissue, cells were incubated with anti-THY1 antibody (abcam, ab11155) at 1:30 dilution on ice for 1 h. Flow cytometry was performed on an ACSAria2 (BD bioscience) flow cytometer. Only cells with FITC signals were collected thereafter.

### Cell culture

HAVSMCs were incubated in SMCM (ScienCell, Cat. 1101) supplemented with 10% FBS, 1% smooth muscle cell growth supplement (SMCGS) and 1% penicillin/streptomycin. Human AD-FB2 cells, harvested from flow cytometry, were cultured in DMEM/F12 medium (Gibco, Cat. 11320033) supplemented with 10% FBS and 1% penicillin/streptomycin. One day before co-culture, HAVSMCs were digested by 0.25% trypsin and seeded in 6-well plates at 1×10^5^ cells per well. After 48 h of culture, the medium (FB2-conditioned medium) was collected, and used for HAVSMC culture for another 48 h. DMEM/F12 supplemented with 5% FBS was used as control medium.

### ELISA

5 mL of venous blood was collected from each volunteer on admission into EDTA tubes (BD, 367863). Blood samples were centrifuged at 4 °C, 1000 × *g* for 15 min. Then, plasma was collected and stored at -80 °C. The levels of human ADAM12 and COL5A1 were examined via ELISA kits (Proteintech, Cat. KE00058; CUSABIO, Cat. CSB-E13447h) according to the manufacturer's instruction. Briefly, 100 μL standards or samples were added into 96-well plates. After incubation at 37°C for 2 h, biotin-conjugated antibody solution, HRP-avidin solution, TMB substrate solution and stop solution were added to each well sequentially. Absorbance at 450 nm was measured within 5 min. Results were calculated by using Curve Expert Professional v1.3.

## Statistics

### Single-cell RNA-Sequencing

#### Raw read processing and mapping

Raw reads were processed by the perl pipeline script which was originally described by Leonard D. Goldstein, et al. 32. First, the validity of reads was checked, and only read pairs whose read 1 uniquely mapped the pre-defined barcode tag (10 nucleotides) and unique molecular identifier (UMI; 11-14 nucleotides) were considered valid. Then, read pairs were filtered by cutadapt (v1.8.1), with parameters: -m 20 --trim-n --max-n 0.7 -q 20. Further, the reads were aligned to genomes of *E.coli*, mycoplasma, yeast, and adapter sequences by bowtie2 (v2.2.4) 33, and all contaminants were filtered by fastq screen (v0.5.1. 4). Clean reads were mapped to the UCSC hg38 genome via STAR (v2.5.2b) 34 and assigned to Ensembl genes 35 by featureCounts (subread-1.4.6-p1 command line) 36. Sequencing reads were further filtered and sorted by a custom barcode filtering pipeline.

#### Cell filtering and data normalization

Cell filtering steps were described in our earlier work 30, 37, 38. The number of captured transcripts per gene was inferred based on UMIs, and read pairs with UMIs containing Ns were excluded. UMIs were limited to 2 s.d. from the mean of log10 UMI of all cells. Further, only cells with a minimally detected gene number of 1000 were kept for downstream analysis. To remove background signal, only genes that were detected in at least 10 cells were considered as passing the threshold. In addition, reads mapped to all 37 mitochondrial genes were excluded (Table S23). Then, as illustrated by Butler et al. 39, per-gene transcript counts were normalized across cells. Within each single cell, the UMI count of each gene was divided by the total UMI number of that cell, and multiplied by size factor 10,000 to obtain a TPM-like value, which was then transformed to natural logarithm.

#### Single cell clustering and annotation of cell state clusters

A total of 3,199 cells were passed our filtering criteria as described above. Clustering was conducted using the Seurat package (v2.3.4) 40. To avoid potential batch effects among subjects and experiments, CCA in Seurat was used to align the subjects and experiments (chips) 39. A Seurat object was conducted for cells of each chip, and nUMI was regressed out using the Scale function. Variable genes among cells were then selected according to their average expression level and dispersion: average log TPM-like value was restricted to between 0.01 and 3 and dispersion was set to greater than 1. Then the MultiCCA function was used with the parameter num.cc = 20 for data alignment. We observed that the first twenty canonical correlation vectors could explain the majority of correlation strengths, and were therefore used for subsequent cell clustering. Main cell types, including endothelial cells (EC), fibroblasts (FB), macrophages (MP), granulocytes (GR) and vascular smooth muscle cell (SMC), and T cells, were then determined according to the expression of canonical molecular markers, which are used to define cell types in previous literatures (Table S24) 37, 41-43.

To address whether there was systematic batch effect in our data, we selected 107 house-keeping genes including 36 transcription elongation factor genes, 15 ribosomal genes, 19 RNA polymerase II subunit genes, and 37 tRNA genes (Table S1). PCA analysis of the housekeeping genes showed that all samples were mixed. From the *t*-SNE result, all clusters contained cells from multiple subjects. Hence, the difference among cell clusters represented true biological difference rather than systematic technical issues.

#### Identification of variable genes

As described by Butler et al. 39, the FindAllMarkers function was used to identify variable genes for clarifying the identity genes of each group of cells, and the FindMarkers function was used for identifying the DEGs between specific two groups of cells. Genes were filtered with a q value of 0.05.

#### Analysis of cell trajectory

Monocle2 (v2.6.0) 44 was used to study pseudotime trajectories of cells. The UMI matrix was used as input and variable genes detected by Seurat were used for building traces. Branches in the cell trajectory represent cells that have alternative gene expression patterns.

#### Analysis of cell similarity

For cell similarity calculation: 1) the average gene expression profiles of cells of different types were calculated. 2) Pearson correlation coefficient of each cell to the cell type's average expression vector was calculated and shown in a violin plot.

#### Cell-cell interaction network

Human secreted proteins were extracted from the CellPhoneDB database. A total of 757 genes were retrieved as secreted genes. We used the FindAllMarkers function in Seurat to define specifically expressed genes in each cluster. Genes encoding secreted protein were selected as possible ligands. To discover possible secreted proteins that directly influence change of SMC along its pseudotime trajectory, we used a cross-match method. First, the GO terms associated with the secreted proteins of each cluster were retrieved by the function of bitr in clusterProfiler (v3.8.1). Then the terms were overlapped with the enrichment result of branching DEGs in the SMC trajectory. Only overlapping terms and associated ligands were kept. To evaluate the total secreting capacity of each cell cluster, the sum of scaled expression (*z*-score) of all specific secreted proteins of each cell cluster was calculated, and the cell clusters were ordered by their *z*-score sum of all specific secreted proteins.

#### Ligand-receptor interaction

Human membrane receptors were extracted from the CellPhoneDB database. A total of 678 genes were retrieved as membrane receptors genes. Within each SMC cluster, membrane receptors were selected according to cluster specific genes. Further, all potential pairs of ligand-receptor interactions were identified, according to a pre-built protein-protein interaction library. Expression of interaction pairs between two clusters was calculated as the product of mean expression of the ligand in the ligand-producing cluster and the mean expression of the receptor in the receptor-producing cluster.

### Bulk RNA-Sequencing

#### Identification of differentially expressed genes

For bulk RNA-Seq analysis, reads were aligned to genomes of hg38 by subread (R version 1.24.2) 45. Uniq reads were kept and then assigned to in-build refseq gene annotation of rsubread using featureCounts. Genes were further filtered, and only those with rpkm>1 in two or more than two samples were kept for differential analysis, as described by Law et al. 46. Differential analysis was conducted with limma 47. Genes with an adjusted *p* value < 0.05 and an absolute logFC value > 0.58 were taken as significantly differentially expressed genes.

### Statistical Analysis

All results are expressed as means ± SD. Student's *t*-test or Wilcoxon rank-sum test were used for comparison of 2 groups as indicated, and one-way ANOVA, then by post hoc analysis was used for comparison of 3 or more groups as indicated in the manuscript. A *p* value < 0.05 was considered statistically significant.

## Results

### Characterization of cellular composition and spatial distribution of human thoracic aorta at single-cell resolution

**Figure 1:**
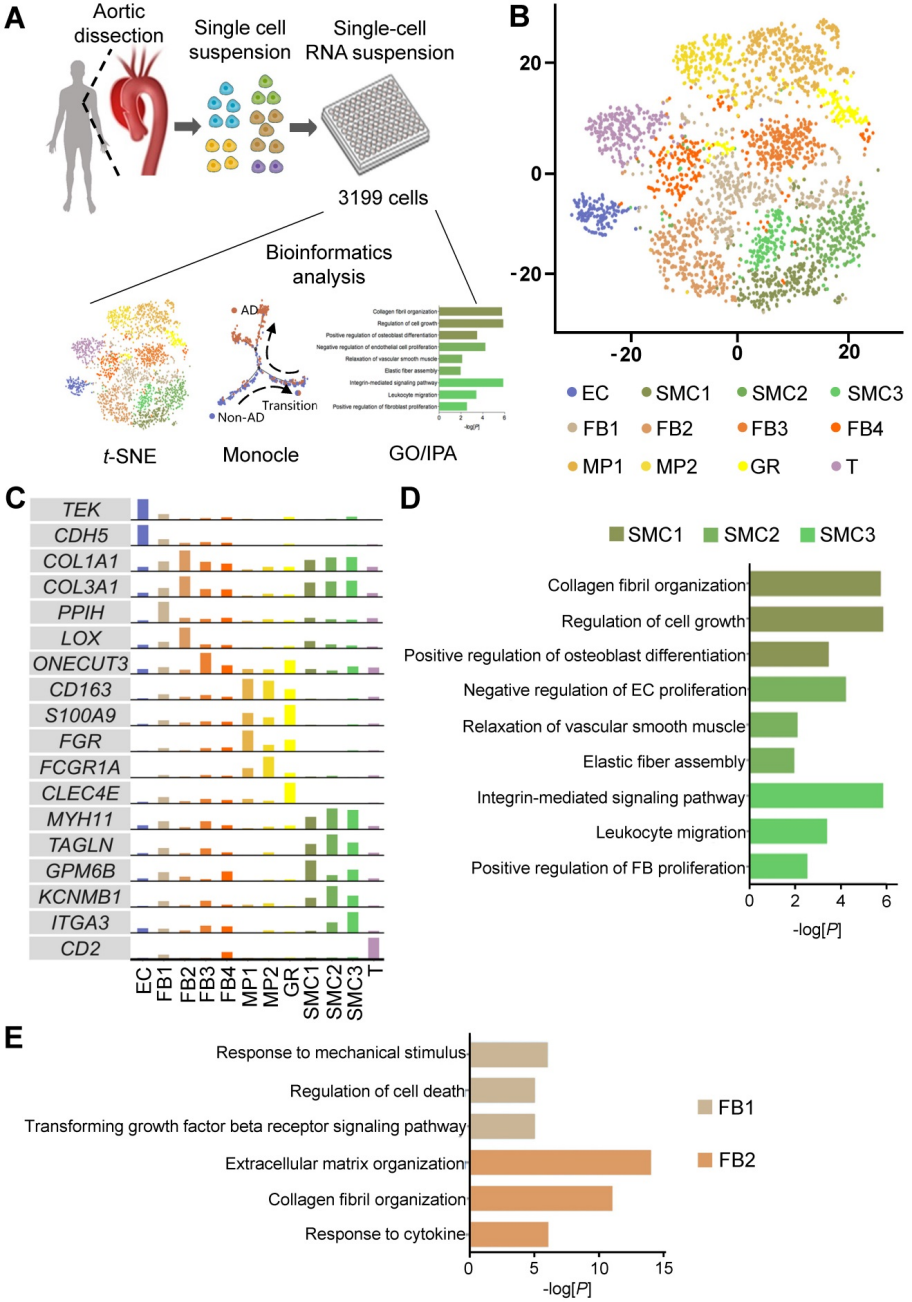
** Characterization of cellular composition of human thoracic aorta at single-cell resolution. (A)** Schematic of study design.** (B)** *t*-distributed stochastic neighbor embedding (*t*-SNE) clustering of 3,199 cells isolated from human Non-AD or AD aortas. **(C)** Expression of canonical markers and specific marker in cell clusters. The cell clusters in (B) were identified by the expression of canonical marker genes. **(D, E)** Gene ontology (GO) analysis of genes specifically expressed in SMCs clusters (D) and FBs clusters (E). Selected top categories are shown.

To investigate the cellular composition of human thoracic aorta, we performed scRNA-seq on cells from the thoracic aortas of 4 Stanford type A aortic dissection (AD) patients without familial thoracic aortic disease and 3 non-AD donors (Non-AD) (Figure 1A, Figure S1A). Upon stringent quality control, 3,199 cells were subjected to further analysis, with a median depth of 182,418 reads / cell, 85% alignment rate / cell, and 1,326 genes / cell (Figure S1B-C). Cells were partitioned with *t*-distributed stochastic neighbor embedding (*t*-SNE) into six major cell types, including endothelial cells (ECs), vascular smooth muscle cells (SMCs), fibroblasts (FBs), macrophages (MPs), granulocytes (GRs) and T cells (T), according to their canonical molecular markers and particular transcriptional signatures (Figure 1B-C, Figure S2). Noteworthily, main cell types, including SMCs, FBs, and MPs, contained multiple subtypes with distinct molecular features (Figure 1C, Figure S2). Possible batch or individual effects were ruled out by the relatively even expression of house-keeping genes, as well as multiple donor sources for each cell subtype (Figure S3A-D; Table S1). The cellular subtypes exhibited functional diversity, as revealed by Gene Ontology (GO) enrichment analysis. For example, SMC subtype 1 (SMC1) displayed functions related to collagen fibril organization, SMC2 and SMC3 were implicated in crosstalk with endothelial cells and fibroblasts, respectively (Figure 1D; Table S2-S4). Similarly, FB1 was linked to response to mechanical stimulus, whereas FB2 was enriched in functions associated with extracellular matrix organization (Figure 1E; Table S5 and S6). In addition, while MP1 was heavily involved in the production of cytokines and inflammatory factors, MP2 showed functional enrichment in the regulation of SMCs (Figure S4; Tables S7 and S8).

**Figure 2:**
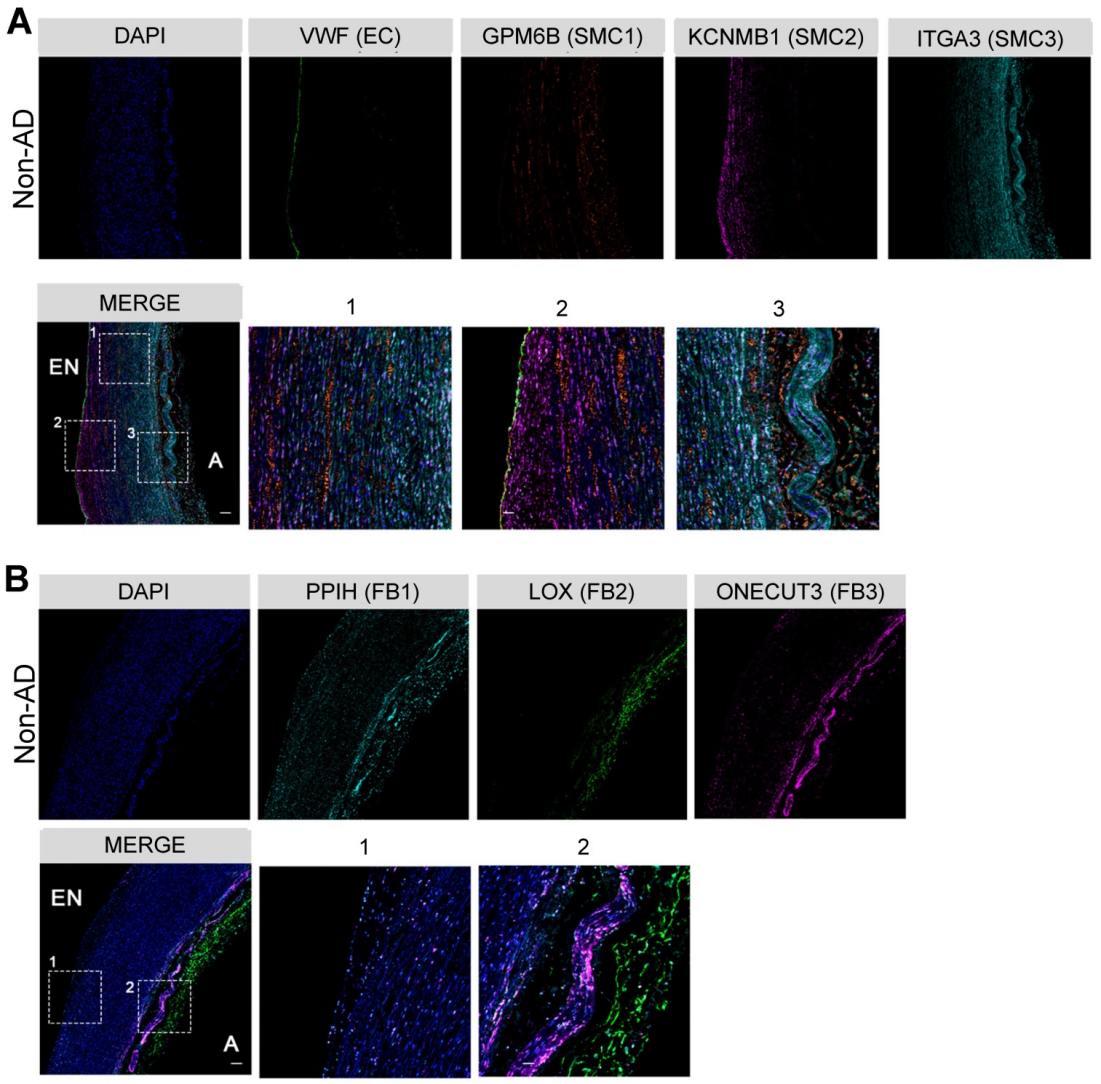
** Characterization of cellular spatial distribution of human thoracic aorta. (A)** Co-staining of EC marker VWF and SMC cluster-specific markers (SMC1, GPM6B; SMC2, KCNMB1; SMC3, ITGA3) in Non-AD sections, zone 1, 2 and 3 in merge are magnified on the right. **(B)** Co-staining of FB cluster-specific markers (FB1, PPIH; FB2, LOX; FB3, ONECUT3) in Non-AD sections, zone 1 and 2 in merge are magnified on the right. Scale bars, 400 μm (top and merge images), 100 μm (magnifications). The images in (A) and (B) represent 3 independent experiments (3 samples each), respectively. EN, endothelium; A, adventitia.

To further understand these functional diversifications, we determined the spatial distributions of the subpopulations of the two major cell types in the artery wall, SMCs and FBs, by multi-color immunohistochemistry (IHC) in non-AD human aorta. As anticipated, VWF^+^ ECs were mainly localized to the endothelium (Figure 2A). While GPM6B^high^ SMC1 distributed across the entire media, KCNMB1^high^ SMC2 and ITGA3^high^ SMC3 displayed preferred localization towards the endothelium and tunica adventitia, respectively (Figure 2A). Interestingly, their localizations were concordant with their functional relevance to collagen organization (SMC1), regulation of ECs (SMC2), and regulation of FBs (SMC3) (Figure 1D). Likewise, FBs clusters showed apparent localization preferences. The FB2 cluster mainly localized to the tunica adventitia, whereas FB1 and FB3 were positioned relatively close to the tunica media, which may facilitate their response to mechanical stimulus and cytokine signaling (Figure 2B). These observations demonstrate the heterogeneity of the cellular composition in the arterial wall, as well as their preferential spatial distributions, suggesting distinct functions of major arterial cell subpopulations.

### Cellular and molecular alterations in human AD

**Figure 3:**
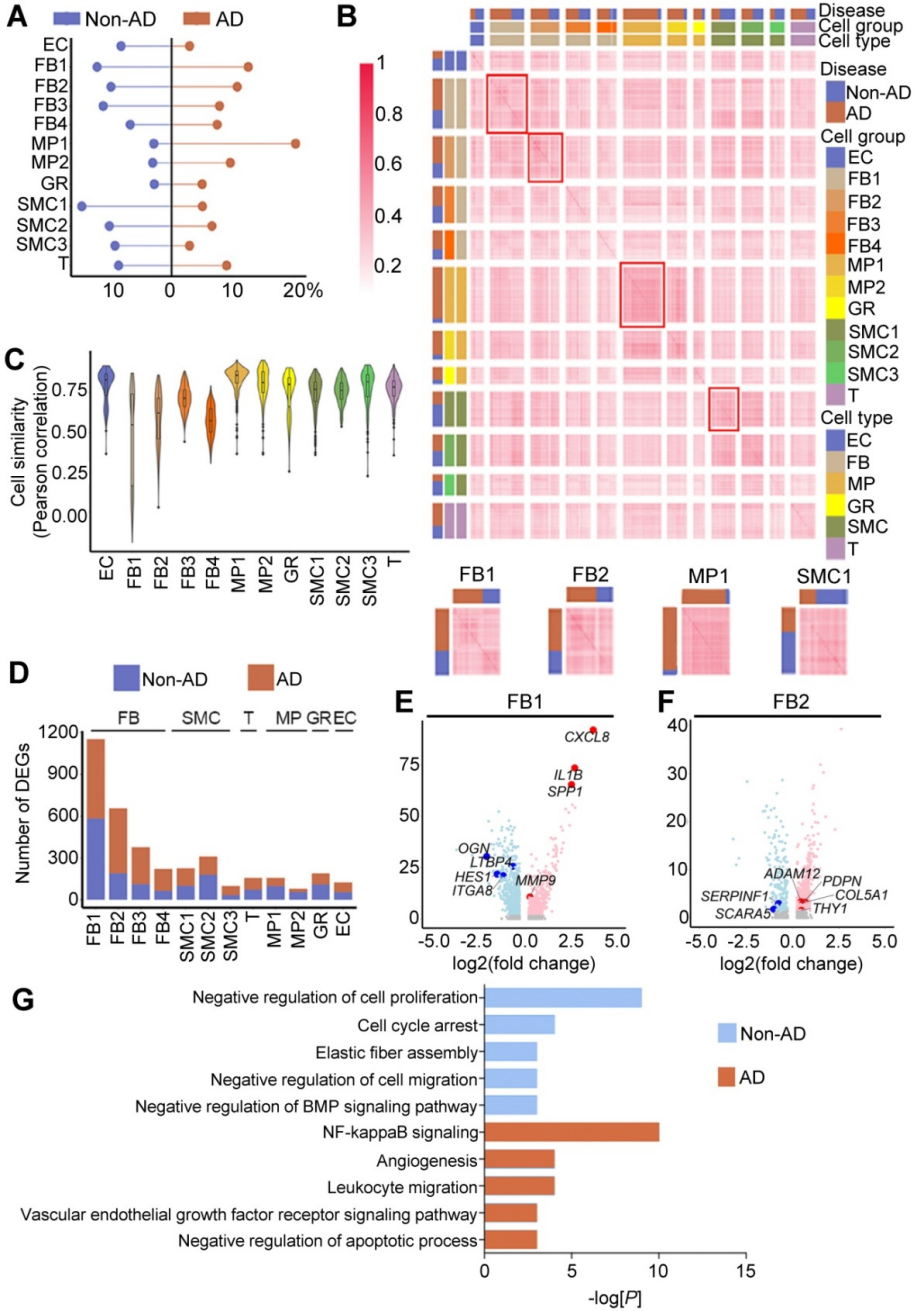
** Cellular and molecular alterations in human AD. (A)** Cell proportion in Non-AD versus AD aortas. The total cell number in one condition was taken as 100%. **(B)** Cell-cell correlation matrices showing correlation coefficients of all pairs of indicated cell clusters. Heatmaps of FB1, FB2, MP1 and SMC1 are magnified on the bottom, showing representative correlation coefficient of these clusters. **(C)** Transcriptome similarities of each cluster, containing both Non-AD and AD cells. The center line shows the median value, the box limits show the upper and lower quartiles. **(D)** Number of differentially expressed genes (DEGs) of each cluster compared between Non-AD and AD states. **(E, F)** Volcano plots to show DEGs in representative clusters, FB1 (E) and FB2 (F). Red indicates up-regulated genes in AD aortas, and blue represents up-regulated genes in Non-AD aortas. (G) Gene ontology analysis of genes specifically expressed in Non-AD FB2 or in AD FB2, selected top categories are shown.

Next, we sought to determine the major cellular changes upon onset of AD. We first compared the cellular compositions of non-AD and AD aortas. In accordance with the previous studies 48, 49, we observed apparent decreases in the abundances of SMCs and ECs, as well as increases in MPs in AD aorta (Figure 3A). However, the proportions of FB subtypes, particularly those of FB1 and FB2, remained largely unchanged (Figure 3A). To gain further insight into the underlying molecular changes in AD pathogenesis, we compared gene expression profiles of these cell clusters, and observed that, compared with other cell types, such as MPs and SMCs, FB subclusters (e.g., FB1 and FB2) formed 2 distinct transcriptomic signatures in AD versus non-AD condition, suggesting state change during AD pathogenesis (Figure 3B). Likewise, FB1 and FB2 exhibited the lowest in-cluster cell similarities compared to other cell clusters (Figure 3C). Indeed, FB1 and FB2 each possessed 2963 (1727 up and 1236 down) and 2541 (1621 up and 920 down) differentially expressed genes (DEGs), respectively, in AD versus non-AD, numbers that were significantly higher than in other cell clusters (Figure 3D, Figure S5; Table S10). Moreover, genes up-regulated in FBs in AD were involved in inflammation (e.g., *IL1B* and *CXCL8* in FB1) and extracellular matrix remodeling (e.g., *ADAM12* and *COL5A1* in FB2), two processes that are known to shape AD progression (Figure 3E-F). Indeed, GO analysis showed that genes up-regulated in FB2 in AD were enriched in biological behaviors related to inflammation, whereas down-regulated genes were enriched in functions associated with cell cycle and elastic fiber assembly (Figure 3G; Table S11 and S12). Together, these findings suggested major changes in the cellular composition during AD pathogenesis, as well as potential functional implications of such changes.

### LOX^high^ fibroblast (FB2) is predicted to play a critical role in the initiation of AD

**Figure 4:**
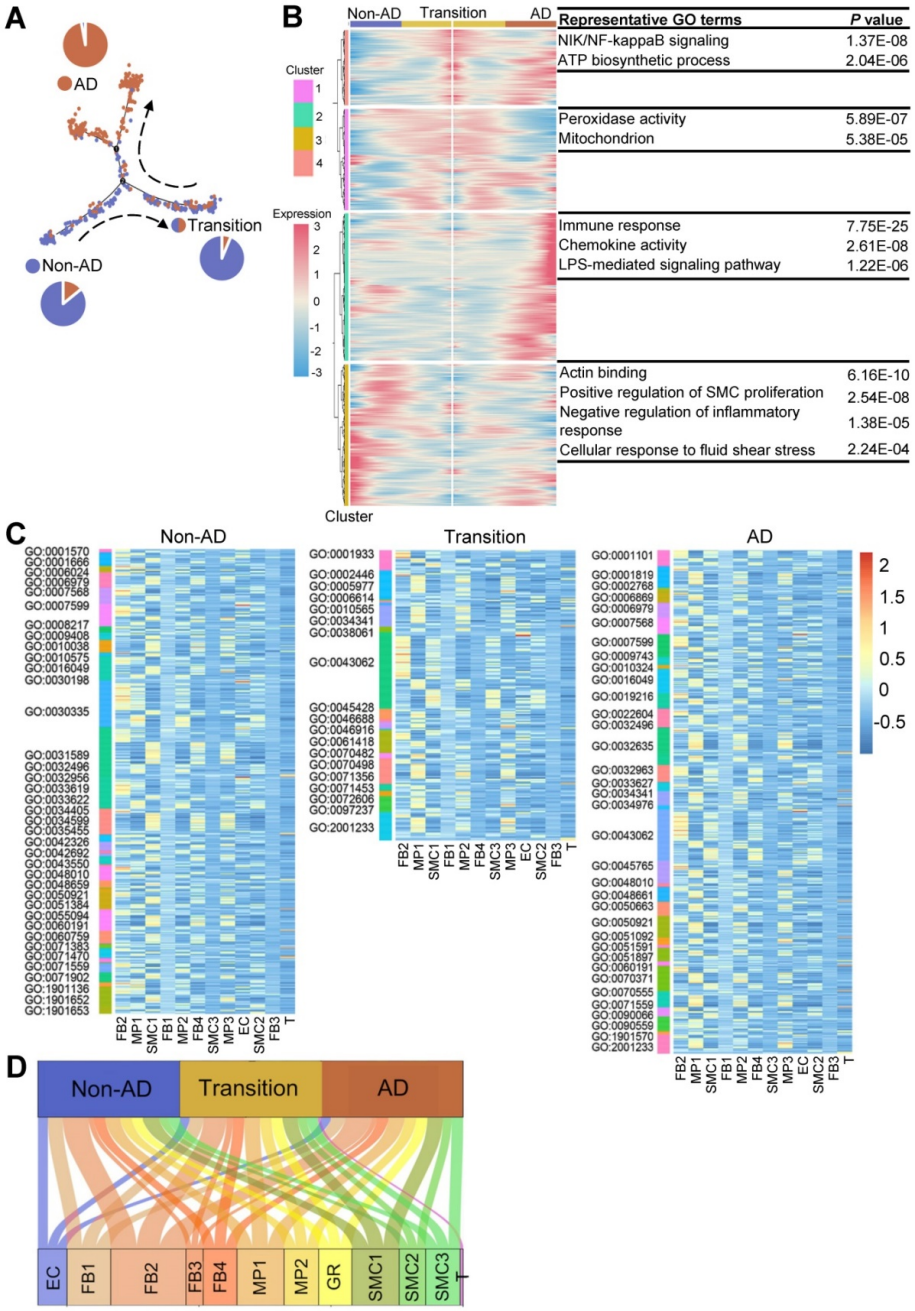
** LOX**^high^** fibroblast (FB2) is predicted to play a critical role in the development of AD. (A)** Monocle analysis showing the ordering of SMCs in pseudotime. Each dot indicates a cell. Each color indicates a cell state. Non-AD represents cells mainly from non-AD arteries; AD, from AD arteries; Transition, mixed cell source from both non-AD and AD. **(B)** Heatmap to display different blocks of top 1,000 differentially expressed genes (DEGs) along the pseudotime trajectory in (A). Selected top gene ontology (GO) terms related to corresponding DEGs are shown on the right. Functional analysis was performed with enrichGO in clusterProfiler, *p* < 0.05 was considered significant enrichment. **(C)** Heatmap to show the quantity of potentially matched pairs between ligands expressed by each cell cluster and SMC signaling pathways at different states (Non-AD, Transition, AD) in pseudotime. **(D)** Sum of all matched ligand-signaling pairs in (C).

The most prominent pathologic feature in AD is the degeneration of the medial layer of the aortic wall, characterized by the loss of SMCs and degradation of the extracellular matrix 5. Therefore, we aligned SMCs in pseudotime to recapitulate its progression trajectory (Figure 4A). SMCs in this trajectory were classified into three states based on the proportions of cell source, termed as non-AD-enriched, transition, or AD-enriched state, respectively. Then, we performed GO analysis using DEGs along the trajectory to analyze biological processes associated with disease progression. Compared to enrichment in energy metabolism and inflammation at transition and AD states, the main molecular features of SMCs during non-AD to transition trajectory comprised regulation of SMC proliferation and regulation of inflammatory response (Figure 4B; Table S13). To explore the roles of cell subpopulations during disease onset and/or progression, we correlated ligands from all cell clusters to GO terms of SMCs in pseudotime.

Compared to other cell clusters, FB2 displayed the highest number of ligands potentially affecting biological processes of SMCs at all states (Figure 4C-D; Table S14-S16). Indeed, there were a number of ligands differentially expressed in FB2 in AD versus non-AD arteries (Figure S6A; Table S17). For instance, ligands such as connective tissue growth factor (CTGF), collagen type V alpha 1 chain (COL5A1) and ADAM Metallopeptidase Domain 12 (ADAM12), were highly expressed in FB2 from AD arteries which were further correlated to multiple biological behaviors of SMCs, reinforcing a critical role of FB2 alteration in SMC pathology and therefore disease progression (Figure S6B). Together with previous observations that FB2 displayed distinct molecular signatures in non-AD versus AD condition (Figure 3), we proposed that alterations of FB2 from a normal state (nFB2) to a diseased state (dFB2) facilitated the initiation of AD.

**Figure 5:**
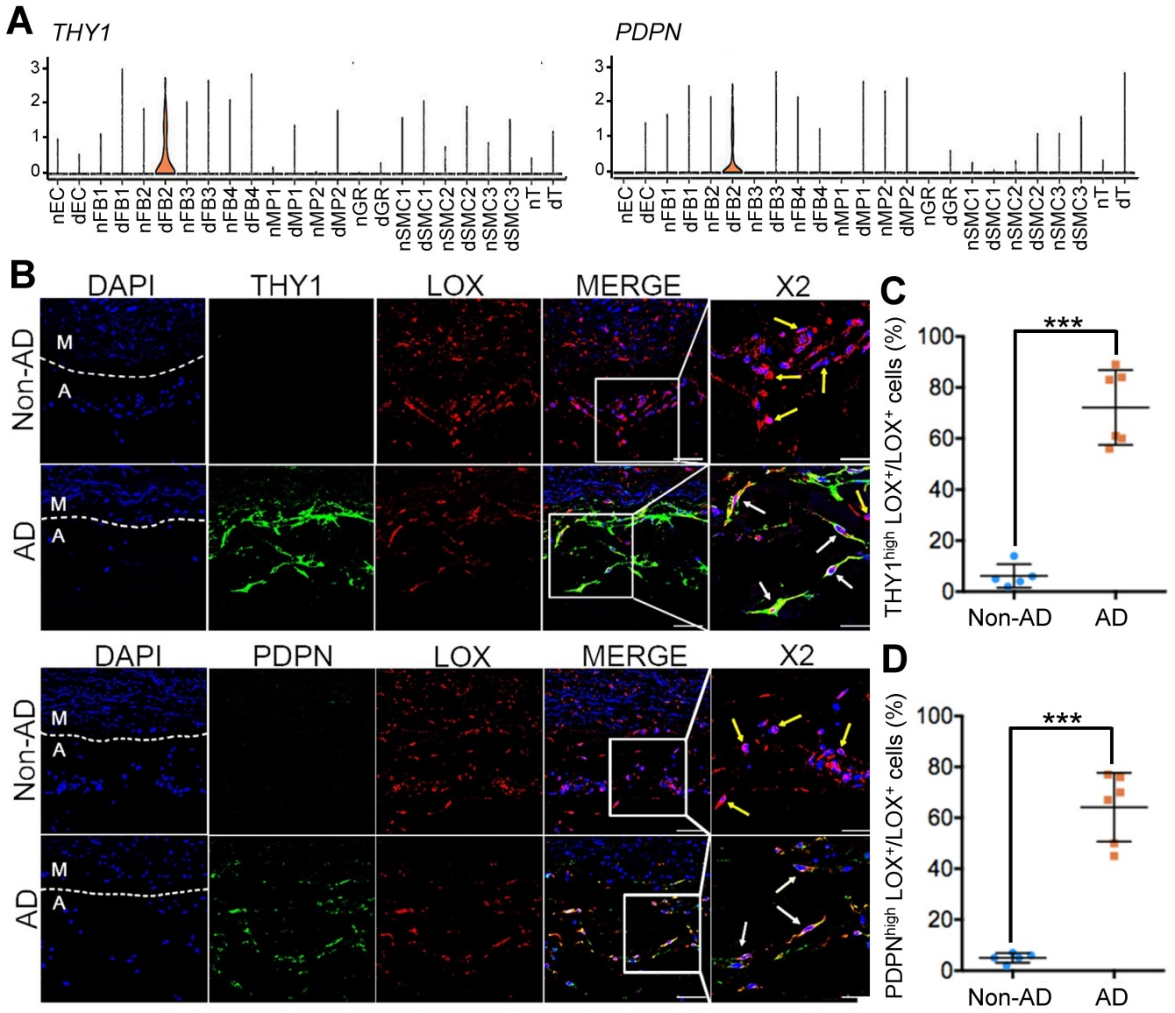
** Expression of dFB2 in human Non-AD and AD samples. (A)** Expression levels of specific markers *THY1* and *PDPN* in diseased FB2 (dFB2), as well as in other clusters based on scRNA-seq data. **(B)** Immunofluorescent staining of FB2 specific marker (LOX) and dFB2 specific markers THY1 or PDPN in human Non-AD or AD sections, respectively. White arrows indicate co-localization, yellow arrows indicate LOX^+^ only cells. Scale bars, 50 μm (left four columns); 25 μm (magnifications). M, media; A, adventitia. The representative images are from 3 and 4 independent experiments (3 samples each), respectively. **(C, D)** Quantitative analysis of THY1^high^LOX^+^ (C) or PDPN^high^LOX^+^ (D) cells in Non-AD versus AD arteries. n = 3 and 5 in Non-AD and AD group, respectively. ***p < 0.001 (Wilcoxon rank-sum test).

**Figure 6:**
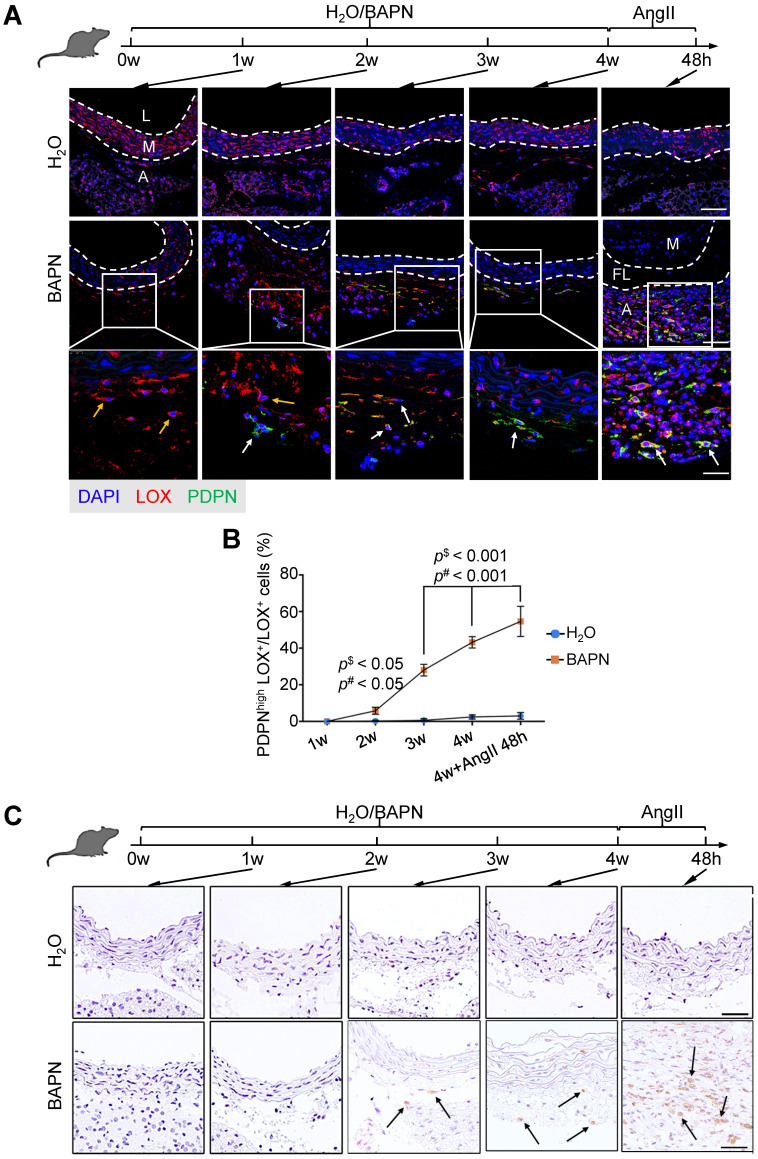
** Expression of dFB2 in mouse model. (A)** Co-staining of FB2 marker LOX and dFB2-specific marker PDPN in mouse thoracic aorta at indicated time points. White arrows indicate co-localization, yellow arrows indicate LOX^+^ only cells. Scale bars, 50 μm (top two images); 25 μm (bottom images). M, media; FL, false lumen; L, lumen; A, adventitia. The representative images are from 3 independent experiments (3 samples each time point). **(B)** Quantitative analysis of PDPN^high^LOX^+^ cells at indicated time points. n = 3. *p*^$^ < 0.05 or 0.001 versus H_2_O group; *p*^#^ < 0.05 or 0.001 versus BAPN group at 1W (One-way ANOVA). **(C)** Immumohistochemical staining of macrophage canonical marker (F4/80) on mice thoracic aorta at indicated time point. Scale bars, 20 μm. The representative images are from 3 independent experiments (3 samples each time point). Black arrows indicate F4/80 positive cells.

To test this hypothesis, we first selected highly expressed genes, *THY1* (Thy-1 Cell Surface Antigen) and *PDPN* (podoplanin), as markers for dFB2 to confirm the observed changes (Figure 5A). THY1 was reported as the marker of activated fibroblasts in heart 50. PDPN is commonly detected in cancer-related fibroblasts 51. These two markers are associated with cell migration, adhesion and differentiation. The specific expression of these two genes may signify high metalloproteinase activity and abnormal ECM synthesis capacity of dFB2. Compared to non-AD arteries, the co-localization of THY1 or PDPN with LOX (lysyl oxidase), a general FB2 marker, raised up from 6% or 5% in non-AD to 72% or 64% in AD, respectively, suggesting a significant increase of dFB2 in AD pathogenesis (Figure 5B-D). Taking it a step further, we used an AD mouse model to elucidate temporal changes of FB2 in AD onset and progression. Mice were administered β-aminopropionitrile (BAPN, 0.5%) for 4 weeks followed by angiotensin II (AngII, 1,000 ng/kg/min) infusion for 48 h to induce AD 52. Interestingly, PDPN^high^ FB2 (dFB2) appeared as early as 2 weeks post administration of BAPN, and continually increased during disease progression (Figure 6A-B). By contrast, MP infiltration was not observed until 3 weeks after BAPN administration (Figure 6C). These results indicated that the appearance of AD-enriched FB2s (dFB2s) may be one of the initiating events inducing changes in SMCs, and subsequently aggravate the disease.

**Figure 7:**
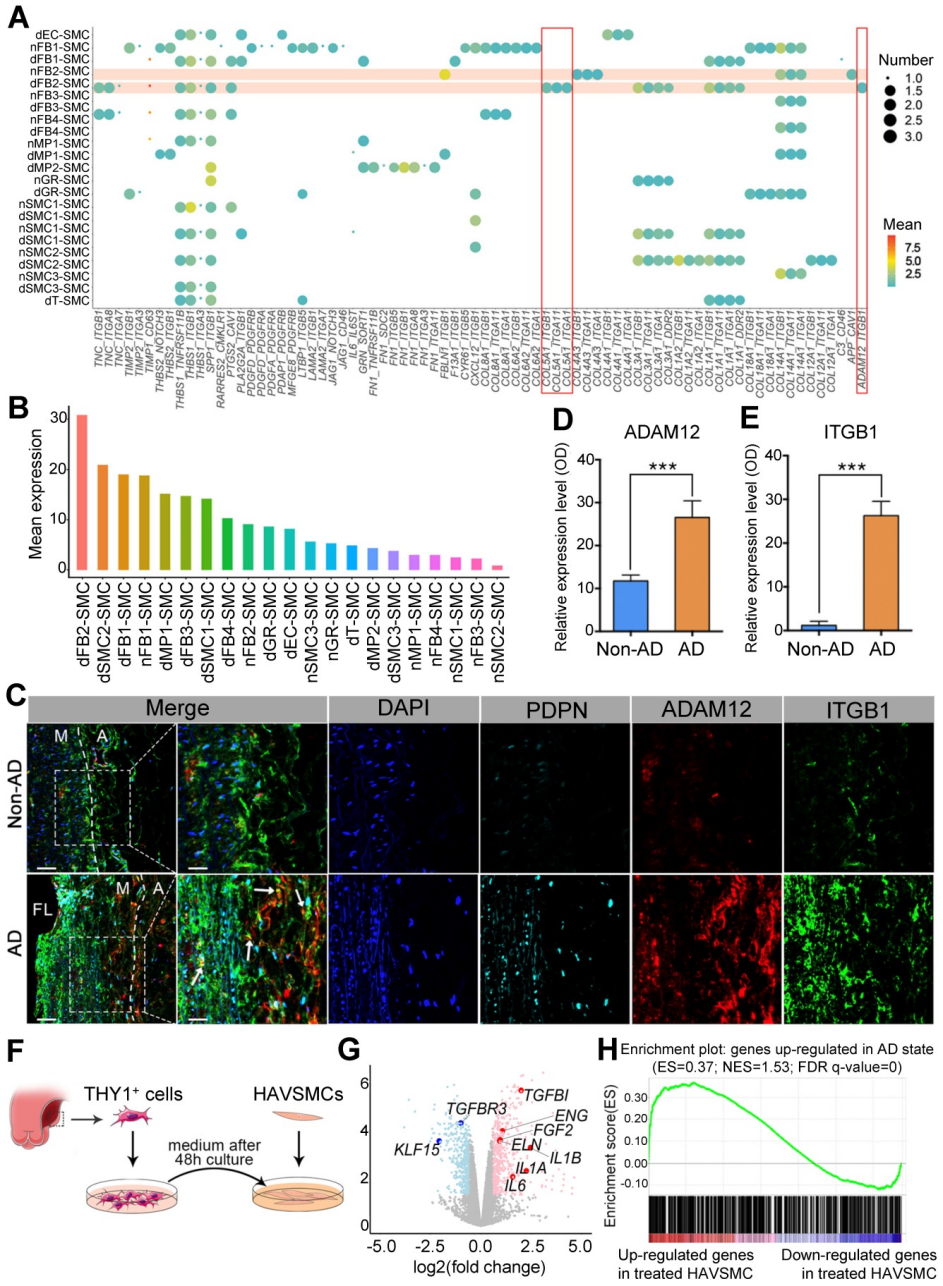
** FB2 affects SMC behaviors via ligand-receptor interaction. (A)** Bubble chart to show putative ligand-receptor pairs between differentially expressed ligands in Non-AD state (n-) or AD state (d-) of each cell cluster and corresponding receptors expressed in SMCs. **(B)** Quantification of ligand-receptor pairs (A) in each cell type. **(C)** Co-staining of dFB2-specific marker PDPN, dFB2-specific ligand ADAM12 and corresponding SMC receptor ITGB1 in human aorta sections. White arrows indicate interaction between ligand and receptor. Scale bars, 100 μm (leftmost column); 50 μm (magnifications). M, media; FL, false lumen; A, adventitia. Representative images are from 3 independent experiments (3 samples each time point). **(D, E)** Quantification of ADAM12 (D) and ITGB1 (E) in (C). n = 3. ***p < 0.001 (Wilcoxon rank-sum test). **(F)** Schematic to show culture of human aortic SMCs (HAVSMCs) with conditioned medium from dFB2. **(G)** Volcano plot to show DEGs in HAVSMCs cultured with conditioned medium. Red and blue colors respectively represent up- and down-regulated genes in treated HAVSMCs cultured in dFB2-conditioned medium. **(H)** Gene Set Enrichment Analysis (GSEA) showing that genes up-regulated in HAVSMCs cultured in conditioned medium were enriched for genes that were highly expressed in AD state.

Next, to investigate how FB2 alterations affected SMCs, we performed ligand-receptor interaction analysis between different cell clusters from non-AD and AD arteries and SMCs (Figure S6C; Table S18), and revealed that FB2 from AD (dFB2) exhibited the most pronounced impacts on SMCs at all stages via ligand-receptor interactions (Figure S7-S9). Surprisingly, the interaction patterns of FB2 and SMCs were drastically different between normal and diseased arteries (Figure 7A). Consistently, dFB2-SMC possessed the highest levels of predicted ligand-receptor interactions among all possible cell-cell interactions (Figure 7B). Careful inspection of all interaction pairs revealed unique pairs between dFB2 and SMCs, exemplified by COL5A1 and ADAM12 and integrin receptors (ITGB1 (integrin subunit beta 1), ITGA1 (integrin subunit alpha 1) and ITGA11 (integrin subunit alpha 11)) (Figure 7A). Multi-color IHC showed that the expression level of ADAM12 in PDPN^high^ FB2 (dFB2) increased nearly 2.5-fold in AD arteries, accompanied by significant increases in ITGB1 in SMCs (Figure 7C-E). These interactions were preferentially observed in the torn and near adventitia regions.

To further demonstrate the functional crosstalk between dFB2 and SMCs, we isolated dFB2 (THY1^+^) from specimens of additional Stanford type A aortic dissection patients by FACS, and then collected conditioned medium to culture normal human aortic SMCs (HAVSMCs) for 48 hours (Figure 7F). The medium from human nFB2 is the most suitable control to elucidate the role of dFB2 on co-cultured SMCs. However, because of the difficulty to collect nFBs from patients due to the limited numbers of donors, we chose HAVSMCs culture medium as an alternative to explore the impact of dFB2 on SMCs in culture. RNA sequencing revealed a number of genes differentially expressed in HAVSMCs upon culture with dFB2-conditioned medium versus control medium (Figure 7G; Table S19). Notably, multiple factors involved in AD, such as *IL1B*, *IL1A*, *IL6*, were significantly induced upon co-culture (Figure 7G). At a global level, gene set enrichment analysis (GSEA) showed that up-regulated genes in HAVSMCs cultured in conditioned medium culture were enriched for genes that were highly expressed in SMCs from AD versus non-AD arteries (Figure 7H). Together, these observations suggested a potential role of dFB2 in the pathological changes of SMCs during AD development via ligand-receptor crosstalk.

### Targeting BMP signaling suppresses onset of AD

**Figure 8:**
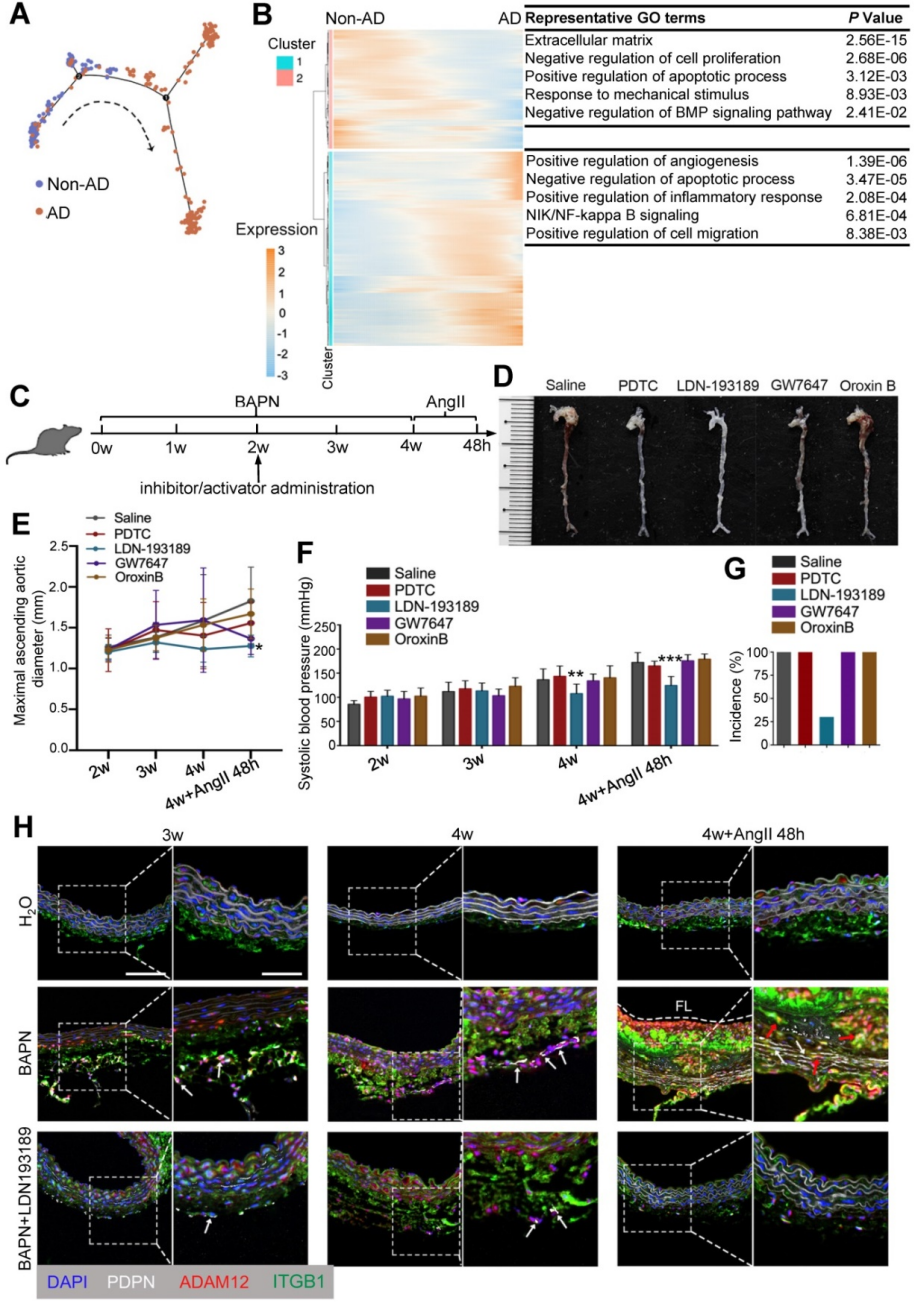
** Targeting BMP signaling suppresses onset of AD in mice. (A)** Monocle analysis showing the ordering of FB2 in pseudotime. Each dot is a cell. Each color indicates a cell state. **(B)** Heatmap to display different blocks of top 1,000 differentially expressed genes (DEGs) along the pseudotime trajectory (A). **(C)** Schematic of *in vivo* intervention experiment showing the administration time point of inhibitors or activators of pathways selected in Figure S8. **(D)** Representative images showing the effects of different compounds on aortas in mouse AD model. **(E)** Maximal ascending aortic diameter in indicated groups. *p < 0.05 compared to Saline group (One-way ANOVA). **(F)** Systolic blood pressure at different time points following treatment. **p < 0.01, ***p < 0.001 compared to Saline group (One-way ANOVA). For D-F, saline, n = 9; PDTC, n = 9; LDN-193189, n = 9; GW7647, n = 9; Oroxin B, n = 9. **(G)** Incidence of aortic dissection following treatment. **(H)** Co-staining of dFB2 marker PDPN, dFB2-specific ligand ADAM12 and corresponding SMC receptor ITGB1in mice aorta sections. White arrows indicate PDPN^high^ cells, red arrows indicate interaction between ADAM12 and ITGB1. Scale bars, 50 μm (left image); 25 μm (magnification). FL, false lumen. Images are representative of 3 independent experiments (3 samples each time point).

Given the pivotal role of FB2 state switching in AD development, we asked whether targeting FB2 was sufficient to prevent AD pathogenesis. To answer this question, we first aligned FB2 in pseudotime to determine molecular basis of state switching (Figure 8A). Gene ontology (GO) analysis on differentially expressed genes (DEGs) along pseudotime revealed a number of pathways potentially involved early FB2 changes, suggesting their involvement in disease onset (Figure 8B; Table S20). To further interrogate signaling pathways driving FB2 alterations, we performed IPA using DEGs between nFB2 and dFB2 to explore potential upstream regulators. Of a total of 7 and 171 pathways that were predicted to be upstream regulators for down-regulated and up-regulated genes, respectively, 3 and 48 pathways were unique to FB2 (Figure S10A-C; Table S21 and S22). Interestingly, a number of pathways were uncovered by both approaches, such as the BMP (bone morphogenetic protein) signaling pathway, a pathway is involved in vascular inflammation and calcification 53, and NF-κB (nuclear factor kappa B) signaling pathway, a pathway that associated with oxidative stress 54. These findings suggested the high confidence of these pathways involved in state switching of FB2. We selected NF-κB signaling, BMP signaling, PPARα and PTEN signaling pathways to investigate the roles of these pathways in FB2 state switching, and used compounds to selectively activate (Oroxin B for PTEN signaling, GW7647 for PPAR activation) or inhibit (PDTC for NF-κB signaling, LDN-193189 for BMP signaling) them in a mouse model of AD (Figure 8C). Compared to other groups, mice treated with BMP signaling inhibitor LDN-193189 displayed reduced maximal ascending aorta diameter and systolic blood pressure (Figure 8D-F). Important, LDN-193189 administration reduced the incidence of AD by 70% (Figure 8G). Mechanistically, we showed that LDN-193189 treatment significantly reduced the number of PDPN^high^ FB2 induced by BAPN (Figure 8H, Figure S10D). In line with this observation, abundant interactions between dFB2 and SMCs, indicated by co-localization of ADAM12 and ITGB1, were detected in BAPN treated mice, whereas LDN-193189 treatment significantly decreased this crosstalk (Figure 8H). Together, these observations suggested that BMP signaling is crucial for the formation of dFB2, and that targeting FB2 state switching effectively reduced AD onset.

### COL5A1 as a candidate circulating marker for AD

**Figure 9:**
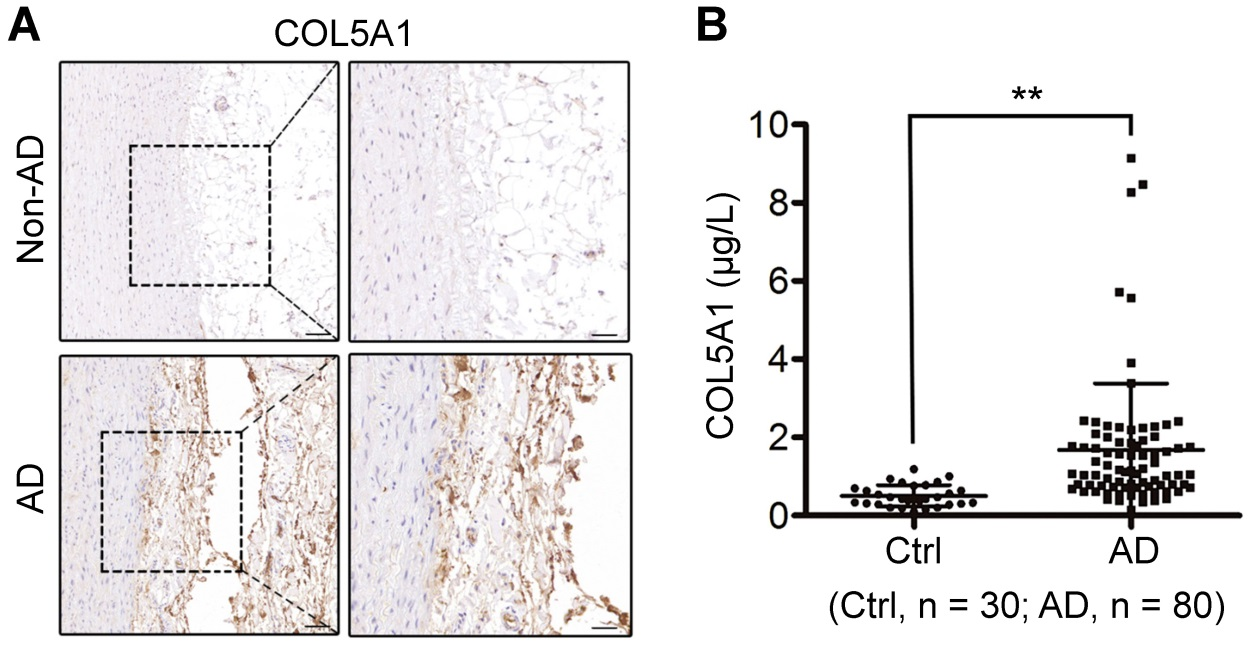
** COL5A1 is a candidate circulating marker for AD. (A)** Immumohistochemical staining of COL5A1 in human thoracic aorta sections. Scale bars, 100 μm (left images), 50 μm (right images). Images are representative of 3 independent experiments. **(B)** Analysis of circulatory COL5A1 levels in human participants. Ctrl, healthy donors; AD, aortic dissection patients. **p < 0.01 (Wilcoxon rank-sum test).

Early diagnosis is critical and lifesaving for appropriate management of AD. Since altered FB2 affected SMC behavior via ligand-receptor interaction, we postulated that ligands secreted by dFB2 could be detected in the circulating blood. Based on single-cell RNA-seq data, ADAM12, COL5A1 and TNC were the ligands specifically expressed by dFB2, and most importantly, they were predicted to affect SMCs via corresponding receptors (Figure 7A, Figure S11). Intriguingly, TNC has already been reported to be a biomarker for predicting in-hospital death from acute AD 55, indicating the reliability of our analysis, as well as feasibility to explore novel biomarkers based on our model. Indeed, consistent with single-cell data, both ADAM12 and COL5A1 were highly expressed in the arterial wall in AD versus non-AD, especially in the tunica adventitia (Figure 7D and 9A). Taking it a step further, we examined the circulating levels of ADAM12 and COL5A1 by ELISA in the plasma from healthy donor (Ctrl), and AD patients (Figure S12). While we failed to detect ADAM12 in both the two groups, the circulating level of COL5A1 was significantly enhanced in the AD group (AD vs Ctrl, *p* < 0.01, Figure 9B), suggesting COL5A1 as a potential diagnostic marker for AD. We examined the circulating levels of COL5A1 in a mouse model of AD (BAPN 4w + AngII 48h), but did not detect changes (data now shown), which may reflect the differences between mouse models and human diseases. This may also arise from the fact that the human samples were from the more advanced stages of AD than the mouse model.

## Discussion

Here, we characterized the cellular composition and their major molecular features of the human aortic wall via single-cell RNA-Seq and *in situ* multi-color staining. In combination with bioinformatic analyses and experimental validation, we unveiled a critical role of FB2 state switching during AD development. Importantly, targeting FB2 state switching by inhibiting BMP signaling pathway significantly suppressed AD onset.

Cell-cell interaction plays a crucial role in physiology and disease, which has been widely studied in development, stem cell and cancer biology 21-24. A previous study revealed stable circuit features in macrophage-fibroblast, suggesting the involvement of fibroblast in homeostasis 56. In our study, we systematically depicted the dynamic alterations of cell-cell interaction in AD onset. Among all putative interactions, FB2 displayed the most pronounced effects on SMC pathogenesis. FBs are the major cell type in many tissues, and are therefore widely involved in homeostasis. Recently, FB subtype switching had been observed in cardiac maturation and hypertrophy, and targeting FB subtype dynamics effectively regulated cardiomyocyte fates, thereby influencing heart function 38, 43. In line with these observations, our study first uncovered the functional crosstalk between dFB2 (THY1^+^) and SMCs via co-culture, then revealed that suppression of FB2 state transition with BMP signaling inhibitor greatly reduced AD pathogenesis, highlighting the possibility to prevent or treat disease by targeting FB-involved cell-cell interactions. Consistent with this notion, a study unraveled interactions between pancreatic stellate cells and pancreatic cancer cells that promoted tumor progression and metastasis, whereas targeting leukaemia inhibitory factor (LIF), a key factor involved in this interaction, markedly slowed tumor progression 22. Collectively, these studies illuminate a path of exploring potential cell state transitions and cell-cell interactions to identify factors critically involved in disease onset and progression.

ECM disorders and SMC rarefaction are common histologic features of AD. For a long time, AD was considered as a disease caused mainly by immune cells and cytokines at the cellular and molecular level. Although plenty of *in vivo* experiments reported the function of immune cells in AD mouse models 55-58, and immune cell markers, such as CD68 and CD4, were detected in human AD specimens, these findings do not demonstrate cellular and molecular aspects other than those of the immune system. To comprehend the molecular foundation unbiasedly, and at a high resolution, we performed single-cell analysis on human AD specimens. We unexpectedly uncovered a crucial role of FB2 state switching in disease progression. Of note, in addition to FB2, other cell subtypes, including MP1, FB1, were also predicted to have certain impacts on AD progression, which warrants further validation.

The adventitia is an essential regulator of vascular wall structure and function. However, its main cell constituent, fibroblasts, did not receive much attention in AD. The composition of adventitial ECM is principally regulated by fibroblasts 61, 62. Similarly, our analysis revealed the strong secretory ability of FB2. The FB2 subcluster from AD patients (dFB2) secreted different ligands compared to non-AD donors, causing aberrant crosstalk between FB2 and SMCs, and finally triggered SMCs pathological changes. Additionally, we demonstrated that the activation of FB2 occurred before macrophage infiltration *in vivo*. Thus, we inferred that immune cells played a role in disease progression, but not initiation. This fibroblast population in AD state expressed higher levels of *THY1* and *PDPN* than in other cell populations. THY1 has been reported to be upregulated in activated fibroblasts and to play vital roles in cell migration, adhesion and differentiation 50. PDPN is commonly found in cancer-related fibroblasts 51. Therefore, these THY1^+^/PDPN^+^/LOX^high^ cells may have higher metalloproteinase activity as well as abnormal ECM synthesis capacity, such that they may contribute to aortic dissection directly via generating disarrayed ECM and enhancing proinflammatory responses. When we treated AD mice with an inhibitor of BMP signaling, the pathway that was specifically activated in dFB2, the incidence rate of AD was significantly lowered. These results support the “outside-in hypothesis”, which indicates that vascular disease is initiated and sustained by the adventitia 61-63. Although BMP inhibition repressed FB2 state transition, how this pathway became activated in FB2 during AD development is still unclear. In addition, as the human samples we used for single-cell RNA-seq were from relatively more advanced stages of AD, they may only represent the consequence of the disease. Further studies are necessary to investigate the roles of FB2 and its state alterations in AD development, and to demonstrate the potential molecular target role of FB2 state alterations in AD prevention and intervention.

Single-cell analysis provides us with a new approach to scrutinize diseases at a higher resolution, which can be particularly useful in identifying potential biomarkers or therapies. At present, a panel of specific biomarkers in the peripheral blood is needed for early diagnosis and classification of AD 64. Here, we found that COL5A1, secreted by FB2, was integral to normal vessel, but was aberrantly accumulated at the dissection zone and in the peripheral blood of AD patients. Thus, it will be worthwhile to robustly validate its use as a biomarker for the early detection of AD.

## Supplementary Material

Supplementary figures.

Supplementary table 1.

Supplementary table 2.

Supplementary table 3.

Supplementary table 4.

Supplementary table 5.

Supplementary table 6.

Supplementary table 7.

Supplementary table 8.

Supplementary table 9.

Supplementary table 10.

Supplementary table 11.

Supplementary table 12.

Supplementary table 13.

Supplementary table 14.

Supplementary table 15.

Supplementary table 16.

Supplementary table 17.

Supplementary table 18.

Supplementary table 19.

Supplementary table 20.

Supplementary table 21.

Supplementary table 22.

Supplementary table 23.

Supplementary table 24.

## Abbreviations

AD: aortic dissection
ADAM12: ADAM metallopeptidase domain 12
AngII: angiotensin II
BAPN: β-aminopropionitrile monofumarate
BMP: bone morphogenetic protein
COL5A1: collagen type V alpha 1 chain
CRP: C-Reactive protein
CTGF: connective tissue growth factor
DEG: differentially expressed genes
dFB2: diseased state FB2
DM: diabetes mellitus
ECM: extracellular matrix
ECs: endothelial cells
ELISA: enzyme-linked immunosorbent assay
FACS: fluorescence-activated cell sorting
FB: fibroblast
FB2: fibroblast subtype 2
FPG: fast plasma glucose
GO: Gene Ontology
GPM6B: glycoprotein M6B
GRs: granulocytes
GSEA: Gene Set Enrichment Analysis
HAVSMCs: human aortic SMCs
HGB: hemoglobin
HR: heart rate
IFN-γ: interferon gamma
IHC: immunohistochemistry
IL-4: interleukin-4
IPA: Ingenuity Pathway Analysis
ITGA1: integrin subunit alpha 1
ITGA3: integrin subunit alpha 3
ITGA11: integrin subunit alpha 11
ITGB1: integrin subunit beta 1
KCNMB1: potassium calcium-activated channel subfamily M regulatory beta subunit 1
LIF: leukaemia inhibitory factor
LOX: lysyl oxidase
MPs: macrophages
nFB2: normal state FB2
NF-κB: nuclear factor kappa B
ONECUT3: one cut homeobox3
PDPN: podoplanin
PPAR: peroxisome proliferator activated receptor
PPIH: peptidylprolyl isomerase H
PTEN: phosphatase and tensin homolog
scRNA-seq: single-cell RNA sequencing
SMC: smooth muscle cell
SMCGS: smooth muscle cell growth supplement
SMCM: smooth muscle cell medium
T: T cell
TNC: tenasin-C
*t*-SNE: *t*-distributed stochastic neighbor embedding
VWF: von Willebrand factor
WBC: white blood cell

## References

[1] An Z, Qiao F, Lu Q, Ma Y, Liu Y, Lu F (2017). Interleukin-6 downregulated vascular smooth muscle cell contractile proteins via ATG4B-mediated autophagy in thoracic aortic dissection. Heart Vessels.

[2] Wang L, Zhang J, Fu W, Guo D, Jiang J, Wang Y (2012). Association of smooth muscle cell phenotypes with extracellular matrix disorders in thoracic aortic dissection. J Vasc Surg.

[3] Del Porto F, di Gioia C, Tritapepe L, Ferri L, Leopizzi M, Nofroni I (2014). The multitasking role of macrophages in stanford type a acute aortic dissection. Cardiology.

[4] Li Y, Ren P, Dawson A, Vasquez HG, Ageedi W, Zhang C (2020). Single-cell transcriptome analysis reveals dynamic cell populations and differential gene expression patterns in control and aneurysmal human aortic tissue. Circulation.

[5] Nienaber CA, Clough RE, Sakalihasan N, Suzuki T, Gibbs R, Mussa F (2016). Aortic dissection. Nat Rev Dis Primers.

[6] Kuhn V, Diederich L, Keller TCS 4th, Kramer CM, Lückstädt W, Panknin C (2017). Red blood cell function and dysfunction: redox regulation, nitric oxide metabolism, anemia. Antioxid Redox Signal.

[7] Ye J, Wang Y, Wang Z, Ji Q, Huang Y, Zeng T (2018). Circulating Th1, Th2, Th9, Th17, Th22, and Treg levels in aortic dissection patients. Mediators Inflamm.

[8] Zeng T, Shi L, Ji Q, Shi Y, Huang Y, Liu Y (2018). Cytokines in aortic dissection. Clin Chim Acta.

[9] Luo F, Zhou X-L, Li J-J, Hui R-T (2009). Inflammatory response is associated with aortic dissection. Ageing Res Rev.

[10] Anzai A, Shimoda M, Endo J, Kohno T, Katsumata Y, Matsuhashi T (2015). Adventitial CXCL1/G-CSF expression in response to acute aortic dissection triggers local neutrophil recruitment and activation leading to aortic rupture. Circ Res.

[11] Kim Y-W, West XZ, Byzova T V (2013). Inflammation and oxidative stress in angiogenesis and vascular disease. J Mol Med (Berl).

[12] Wu Z, Chang J, Ren W, Hu Z, Li B, Liu H (2017). Bindarit reduces the incidence of acute aortic dissection complicated lung injury via modulating NF-κB pathway. Exp Ther Med.

[13] Xia L, Sun C, Zhu H, Zhai M, Zhang L, Jiang L (2020). Melatonin protects against thoracic aortic aneurysm and dissection through SIRT1-dependent regulation of oxidative stress and vascular smooth muscle cell loss. J Pineal Res.

[14] Portelli SS, Hambly BD, Jeremy RW, Robertson EN (2021). Oxidative stress in genetically triggered thoracic aortic aneurysm: role in pathogenesis and therapeutic opportunities. Redox Rep.

[15] Landenhed M, Engström G, Gottsäter A, Caulfield MP, Hedblad B, Newton-Cheh C (2015). Risk profiles for aortic dissection and ruptured or surgically treated aneurysms: a prospective cohort study. J Am Heart Assoc.

[16] Hagan PG, Nienaber CA, Isselbacher EM, Bruckman D, Karavite DJ, Russman PL (2000). The international registry of acute aortic dissection (IRAD): new insights into an old disease. JAMA.

[17] Humphrey JD, Milewicz DM, Tellides G, Schwartz MA (2014). Cell biology. Dysfunctional mechanosensing in aneurysms. Science.

[18] Li R, Yi X, Wei X, Huo B, Guo X, Cheng C (2018). EZH2 inhibits autophagic cell death of aortic vascular smooth muscle cells to affect aortic dissection. Cell Death Dis.

[19] Yan Y, Tan MW, Xue X, Ding XY, Wang GK, Xu ZY (2016). Involvement of Oct4 in the pathogenesis of thoracic aortic dissection via inducing the dedifferentiated phenotype of human aortic smooth muscle cells by directly upregulating KLF5. J Thorac Cardiovasc Surg.

[20] An Z, Liu Y, Song ZG, Tang H, Yuan Y, Xu ZY (2017). Mechanisms of aortic dissection smooth muscle cell phenotype switch. Cardiovasc Surg.

[21] Vento-Tormo R, Efremova M, Botting RA, Turco MY, Vento-Tormo M, Meyer KB (2018). Single-cell reconstruction of the early maternal-fetal interface in humans. Nature.

[22] Shi Y, Gao W, Lytle NK, Huang P, Yuan X, Dann AM (2019). Targeting LIF-mediated paracrine interaction for pancreatic cancer therapy and monitoring. Nature.

[23] Aguirre A, Rubio ME, Gallo V (2010). Notch and EGFR pathway interaction regulates neural stem cell number and self-renewal. Nature.

[24] Tirosh I, Izar B, Prakadan SM, Wadsworth MH 2nd, Treacy D, Trombetta JJ (2016). Dissecting the multicellular ecosystem of metastatic melanoma by single-cell RNA-seq. Science.

[25] Cochain C, Vafadarnejad E, Arampatzi P, Pelisek J, Winkels H, Ley K (2018). Single-cell RNA-Seq reveals the transcriptional landscape and heterogeneity of aortic macrophages in murine atherosclerosis. Circ Res.

[26] Winkels H, Ehinger E, Vassallo M, Buscher K, Dinh HQ, Kobiyama K (2018). Atlas of the immune cell repertoire in mouse atherosclerosis defined by single-cell RNA-sequencing and mass cytometry. Circ Res.

[27] Vanlandewijck M, He L, Mäe MA, Andrae J, Ando K, Del Gaudio F (2018). A molecular atlas of cell types and zonation in the brain vasculature. Nature.

[28] He L, Vanlandewijck M, Mäe MA, Andrae J, Ando K, Del Gaudio F (2018). Single-cell RNA sequencing of mouse brain and lung vascular and vessel-associated cell types. Sci Data.

[29] Li Y, Ren P, Dawson A, Vasquez HG, Ageedi W, Zhang C (2020). Single-cell transcriptome analysis reveals dynamic cell populations and differential gene expression patterns in control and aneurysmal human aortic tissue. Circulation.

[30] Wang L, Yu P, Zhou B, Song J, Li Z, Zhang M (2020). Single-cell reconstruction of the adult human heart during heart failure and recovery reveals the cellular landscape underlying cardiac function. Nat Cell Biol.

[31] Yuan X, Zhang T, Yao F, Liao Y, Liu F, Ren Z (2018). THO complex-dependent posttranscriptional control contributes to vascular smooth muscle cell fate decision. Circ Res.

[32] Goldstein LD, Chen YJ, Dunne J, Mir A, Hubschle H, Guillory J (2017). Massively parallel nanowell-based single-cell gene expression profiling. BMC Genomics.

[33] Langmead B, Salzberg SL (2012). Fast gapped-read alignment with Bowtie 2. Nature Methods.

[34] Dobin A, Davis CA, Schlesinger F, Drenkow J, Zaleski C, Jha S (2013). STAR: ultrafast universal RNA-seq aligner. Bioinformatics.

[35] Zerbino DR, Achuthan P, Akanni W, Amode MR, Barrell D, Bhai J (2018). Ensembl 2018. Nucleic Acids Res.

[36] Liao Y, Smyth GK, Shi W (2014). featureCounts: an efficient general purpose program for assigning sequence reads to genomic features. Bioinformatics.

[37] Yao F, Yu P, Li Y, Yuan X, Li Z, Zhang T (2018). Histone variant H2A.Z is required for the maintenance of smooth muscle cell identity as revealed by single-cell transcriptomics. Circulation.

[38] Ren Z, Yu P, Li D, Li Z, Liao Y, Wang Y (2020). Single-cell reconstruction of progression trajectory reveals intervention principles in pathological cardiac hypertrophy. Circulation.

[39] Butler A, Hoffman P, Smibert P, Papalexi E, Satija R (2018). Integrating single-cell transcriptomic data across different conditions, technologies, and species. Nat Biotechnol.

[40] Satija R, Farrell JA, Gennert D, Schier AF, Regev A (2015). Spatial reconstruction of single-cell gene expression data. Nat Biotechnol.

[41] Fernandez DM, Rahman AH, Fernandez NF, Chudnovskiy A, Amir ED, Amadori L (2019). Single-cell immune landscape of human atherosclerotic plaques. Nat Med.

[42] Zhang W, Zhang S, Yan P, Ren J, Song M, Li J (2020). A single-cell transcriptomic landscape of primate arterial aging. Nat Commun.

[43] Wang Y, Yao F, Wang L, Li Z, Ren Z, Li D (2020). Single-cell analysis of murine fibroblasts identifies neonatal to adult switching that regulates cardiomyocyte maturation. Nat Commun.

[44] Trapnell C, Cacchiarelli D, Grimsby J, Pokharel P, Li S, Morse M (2014). The dynamics and regulators of cell fate decisions are revealed by pseudotemporal ordering of single cells. Nat Biotechnol.

[45] Liao Y, Smyth GK, Shi W (2013). The Subread aligner: fast, accurate and scalable read mapping by seed-and-vote. Nucleic Acids Res.

[46] Law CW, Alhamdoosh M, Su S, Dong X, Tian L, Smyth GK (2016). RNA-seq analysis is easy as 1-2-3 with limma, Glimma and edgeR. F1000Res.

[47] Ritchie ME, Phipson B, Wu D, Hu Y, Law CW, Shi W (2015). limma powers differential expression analyses for RNA-sequencing and microarray studies. Nucleic Acids Res.

[48] Liao WL, Tan MW, Yuan Y, Wang GK, Wang C, Tang H (2015). Brahma-related gene 1 inhibits proliferation and migration of human aortic smooth muscle cells by directly up-regulating Ras-related associated with diabetes in the pathophysiologic processes of aortic dissection. J Thorac Cardiovasc Surg.

[50] Yang J, Zhan XZ, Malola J, Li ZY, Pawar JS, Zhang HT (2020). The multiple roles of Thy-1 in cell differentiation and regeneration. Differentiation.

[51] Cremasco V, Astarita JL, Grauel AL, Keerthivasan S, MacIsaac K, Woodruff MC (2018). FAP delineates heterogeneous and functionally divergent stromal cells in immune-excluded breast tumors. Cancer Immunol Res.

[52] Anzai A, Shimoda M, Endo J, Kohno T, Katsumata Y, Matsuhashi T (2015). Adventitial CXCL1/G-CSF expression in response to acute aortic dissection triggers local neutrophil recruitment and activation leading to aortic rupture. Circ Res.

[53] Cai J, Pardali E, Sánchez-Duffhues G, ten Dijke P (2012). BMP signaling in vascular diseases. FEBS Lett.

[54] Sies H, Berndt C, Jones DP (2017). Oxidative Stress. Annu Rev Biochem.

[55] Guo T, Zhou X, Zhu A, Peng W, Zhong Y, Chai X (2019). The role of serum tenascin-C in predicting in-hospital death in acute aortic dissection. Int Heart J.

[56] Zhou X, Franklin RA, Adler M, Jacox JB, Bailis W, Shyer JA (2018). Circuit design features of a stable two-cell system. Cell.

[57] Hu H, Zhang G, Hu H, Liu W, Liu J, Xin S (2019). Interleukin-18 expression increases in the aorta and plasma of patients with acute aortic dissection. Mediators Inflamm.

[58] Kurihara T, Shimizu-Hirota R, Shimoda M, Adachi T, Shimizu H, Weiss SJ (2012). Neutrophil-derived matrix metalloproteinase 9 triggers acute aortic dissection. Circulation.

[59] Balta S, Alemdar R, Yildirim AO, Erdogan S, Ozturk C, Celik T (2017). The relationship between neutrophil-lymphocyte ratio and acute aortic dissection. Perfusion.

[60] Son BK, Sawaki D, Tomida S, Fujita D, Aizawa K, Aoki H (2015). Granulocyte macrophage colony-stimulating factor is required for aortic dissection/intramural haematoma. Nat Commun.

[61] Tinajero MG, Gotlieb AI (2020). Recent developments in vascular adventitial pathobiology: the dynamic adventitia as a complex regulator of vascular disease. Am J Pathol.

[62] Stenmark KR, Yeager ME, El Kasmi KC, Nozik-Grayck E, Gerasimovskaya EV, Li M (2013). The adventitia: essential regulator of vascular wall structure and function. Annu Rev Physiol.

[63] Maiellaro K, Taylor WR (2007). The role of the adventitia in vascular inflammation. Cardiovasc Res.

[64] Morello F, Piler P, Novak M, Kruzliak P (2014). Biomarkers for diagnosis and prognostic stratification of aortic dissection: challenges and perspectives. Biomark Med.

