# Supplementary material for: Dysregulation of interaction between LOXhigh fibroblast and smooth muscle cells contributes to the pathogenesis of aortic dissection
Source: Theranostics. 2022 Jan 1;12(2):910–28. doi: 10.7150/thno.66059 (PMC8692905; doi:10.7150/thno.66059)
Supplement: Supplementary file 1 — Supplementary figures. [file thnov12p0910s1.pdf]

Supplemental Materials for

Dysregulation of Interaction Between Lox^{High} Fibroblast and Smooth Muscle Cells Contributes to the Pathogenesis of Aortic Dissection

Yinan Chen, Tao Zhang, Fang Yao, Xiang Gao, Dandan Li, Shufang Fu, Lin Mao, Fei Liu, Xuelin Zhang, Yongle Xu, Jianqing Deng, Weihao Li, Guangpu Fan, Cangsong Xiao, Yu Chen, Li Wang, * Wei Guo,* Bingying Zhou*

*Corresponding authors. Email: zhouby@fuwai.pumc.edu.cn, pla301dml@vip.sina.com, wangl@pumc.edu.cn.

A

	Non-AD1	Non-AD2	Non-AD3	AD1	AD2	AD3	AD4
**Sex**	M	M	M	M	F	M	M
**Age**	25	72	54	51	38	32	65
**Diagnosis**	Dilated cardiomyopathy	Coronary heart disease	Coronary heart disease	Stanford type A aortic dissection	Stanford type A aortic dissection	Stanford type A aortic dissection	Stanford type A aortic dissection
**Aortic diameter (mm)**	22.7	32	35	65	41.51	58.27	58.31
**Location of dissection**	No	No	No	Ascending aorta, aortic arch, descending aorta, part of abdominal aorta	Ascending aorta, aortic arch, descending aorta	Ascending aorta, aortic arch, descending aorta, part of abdominal aorta	Ascending aorta, aortic arch, descending aorta, part of abdominal aorta
**Smoking**	No	No	Yes	Yes	No	No	No
**Alcohol**	No	No	Yes	No	No	No	No
**Diabetes mellitus**	No	No	Yes	No	No	No	No
**Hypertension**	Yes	No	Yes	Yes	Yes	No	Yes
**Systolic pressure (mmHg)**	148	112	138	213	146	120	181
**Diastolic pressure (mmHg)**	91	87	66	130	89	65	80

B

	single cell RNA-Seq						
**Condition**	**Control**			**Patients**			
**Total cells**	1,198 (n = 3)			2,001 (n = 4)			
Subjects	Non-AD1	Non-AD2	Non-AD3	AD1	AD2	AD3	AD4
Cell number	759	74	365	441	337	562	661

C

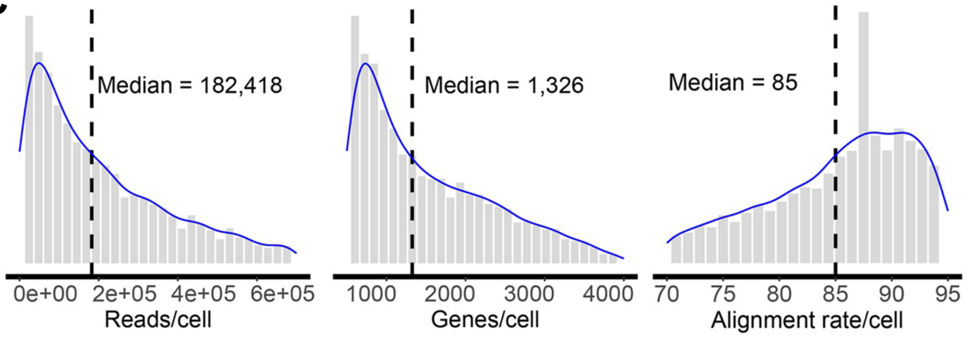

Supplemental figure 1. Quality control of single-cell RNA-Seq. (A) Patient information for single-cell RNA-sequencing samples. **(B)** Cell numbers collected from each donor after quality control filtering. **(C)** Quality metrics for single-cell RNA-seq data showing the distributions of number of reads, number of genes detected, and alignment rate per cell.

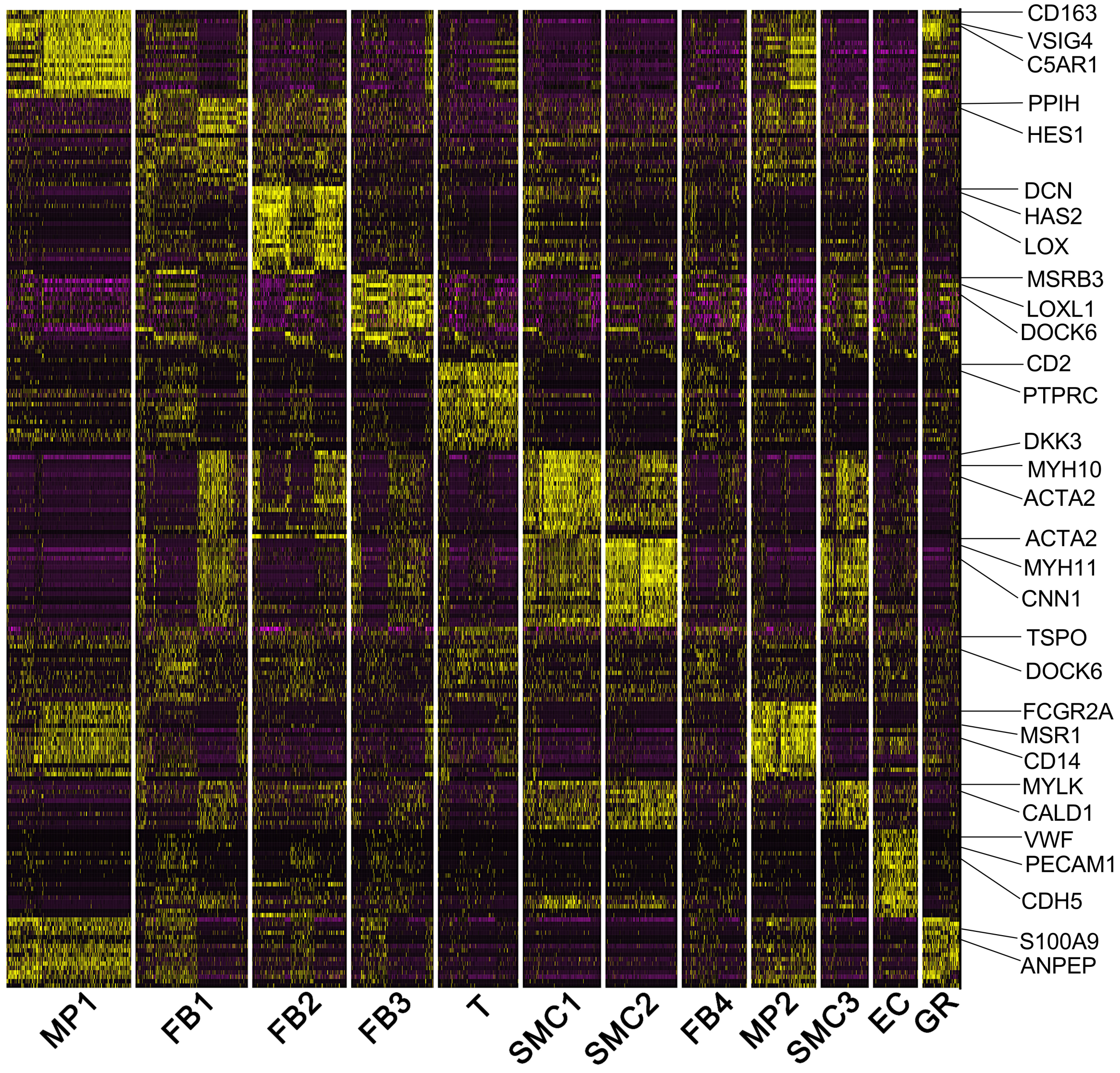

Supplemental figure 2. Heatmap showing particular transcriptional signatures of each cell cluster and the top expressed genes in each cluster (complete DEGs shown in Table S24).

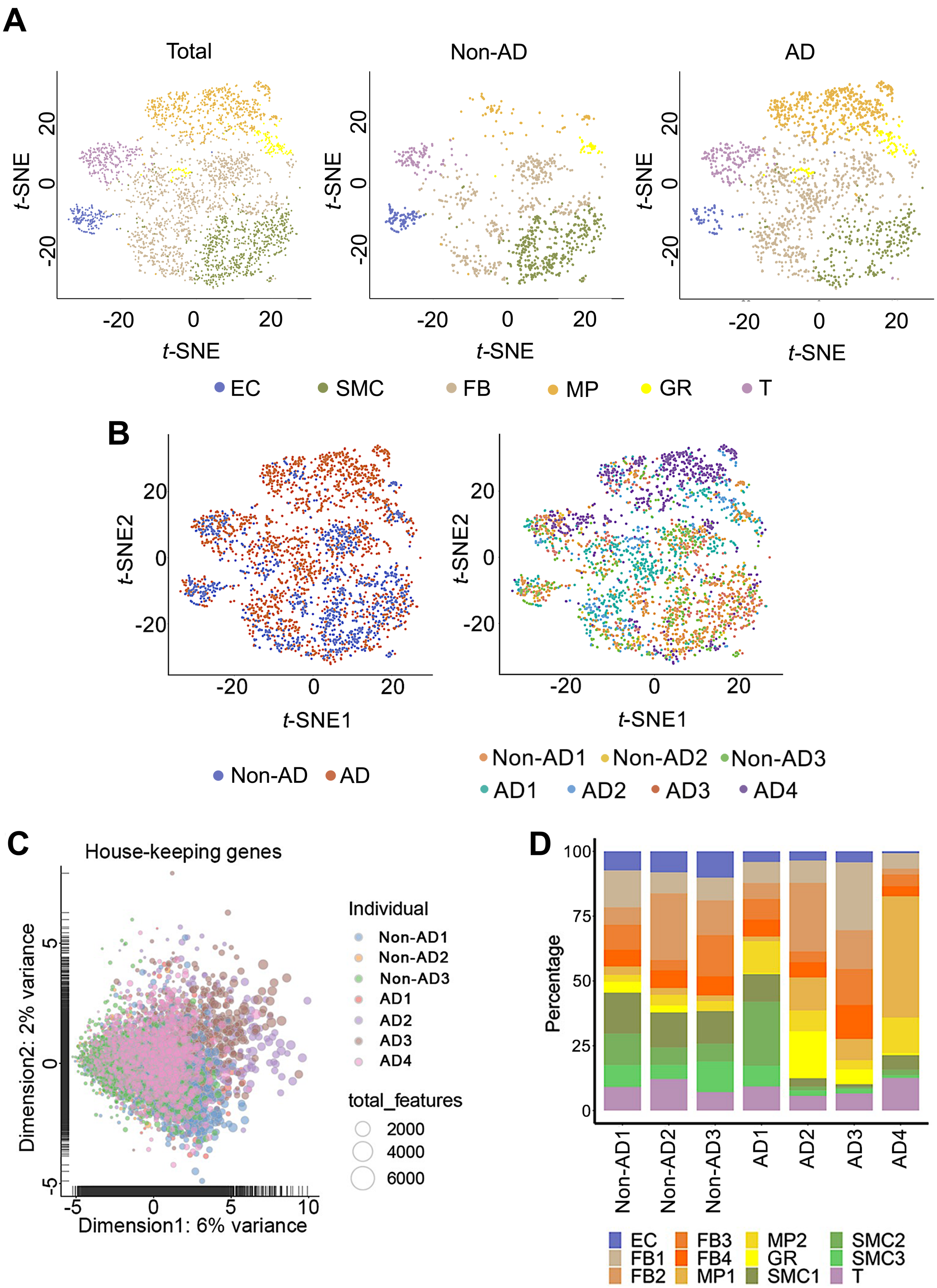

Supplemental figure 3. Quality control of single-cell RNA-Seq. (A) *t*-SNE clustering of 3,199 cells isolated from normal or diseased aortas. **(B)** Cells were marked by disease state or subjects,

respectively. **(C)** Cell clustering using house-keeping genes. **(D)** Proportion of clusters in each subject.

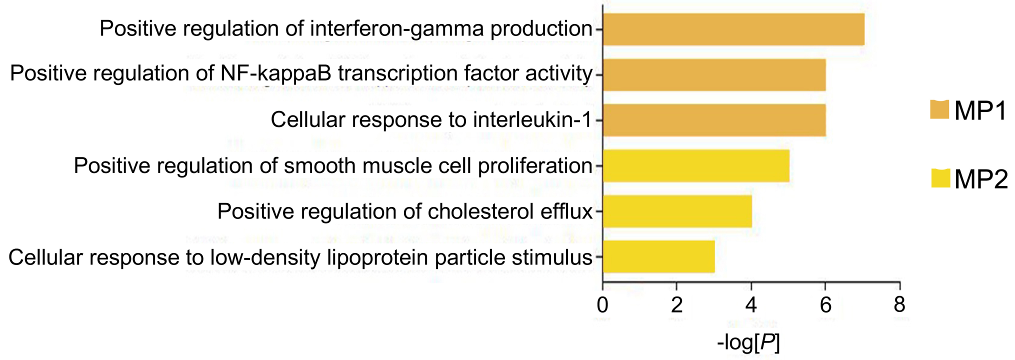

Supplemental figure 4. Gene ontology analysis of genes specifically expressed in MP clusters.

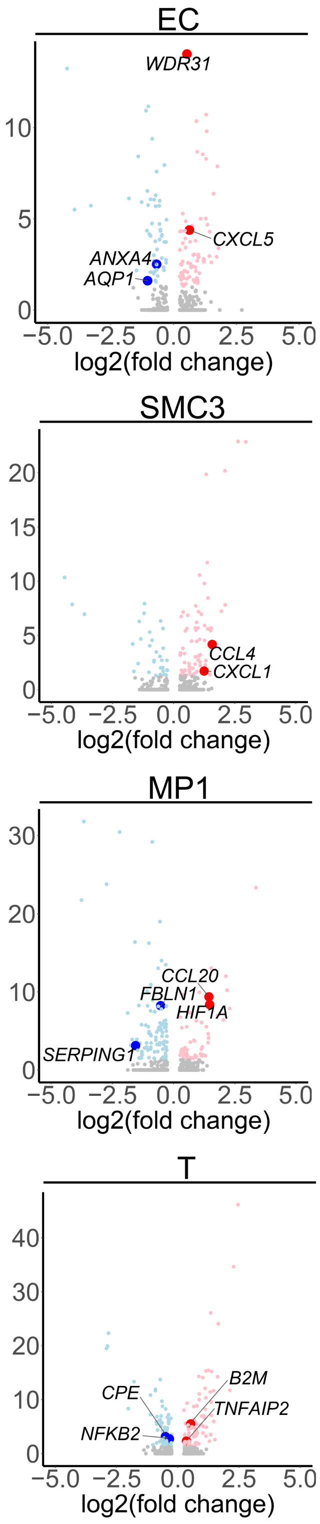

Supplemental figure 5. Molecular alterations in human AD. Volcano plot to show DEGs in indicated cell clusters. Red color indicates up-regulated genes in AD aortas, and blue color represents up-regulated genes in Non-AD aortas.

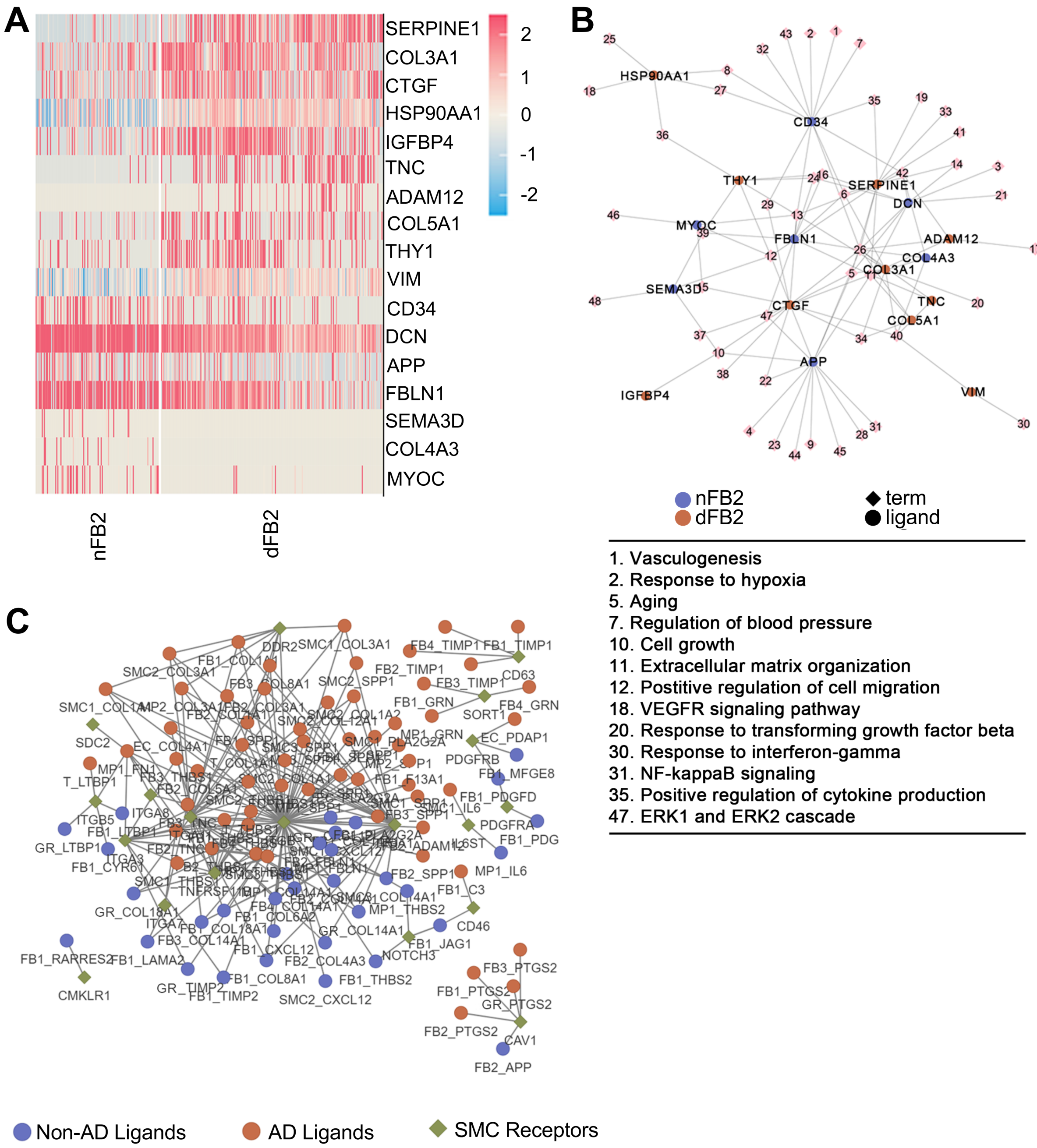

Supplemental figure 6. Putative cell-cell interactions in AD versus Non-AD. (A) Heatmap to show differentially expressed ligands in diseased FB2 (dFB2) versus normal FB2 (nFB2). **(B)** Correlation analysis to show potentially matched pairs between the ligands secreted by FB2 of distinct states (nFB2 and dFB2) and signaling pathways in SMCs. Each circle/dot indicates a

ligand expressed by FB2 of certain states. Each pink diamond represents signaling pathway of SMCs. Each connecting line indicates the correlation between a ligand and a specific pathway. **(C)** Correlation analysis to show potentially matched pairs between ligands expressed by indicated cell clusters and receptors expressed by SMCs. Each circle/dot indicates a ligand expressed by a cluster of a certain state. Each green diamond represents a receptor on SMCs. Each connecting line indicates the correlation between a ligand and a receptor.

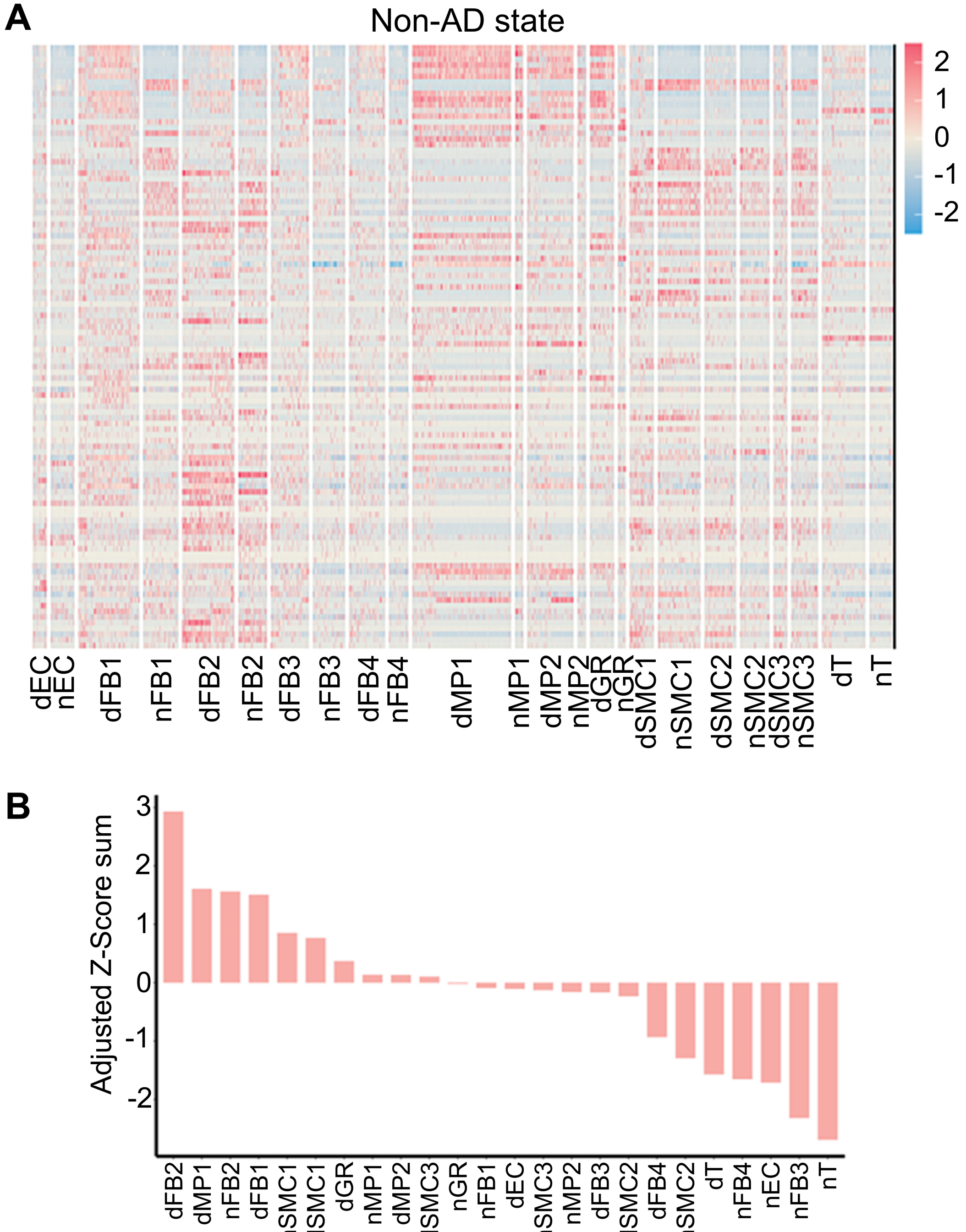

Supplemental figure 7. Quantification of putative cell-cell interactions in AD versus Non-AD. (A) Heatmap to show potential pairs between ligands (Supplemental figure 6C) expressed by indicated cell clusters and signaling pathways in Non-AD state SMCs at in Monocle analysis. **(B)** Sum of all matched pairs in (A).

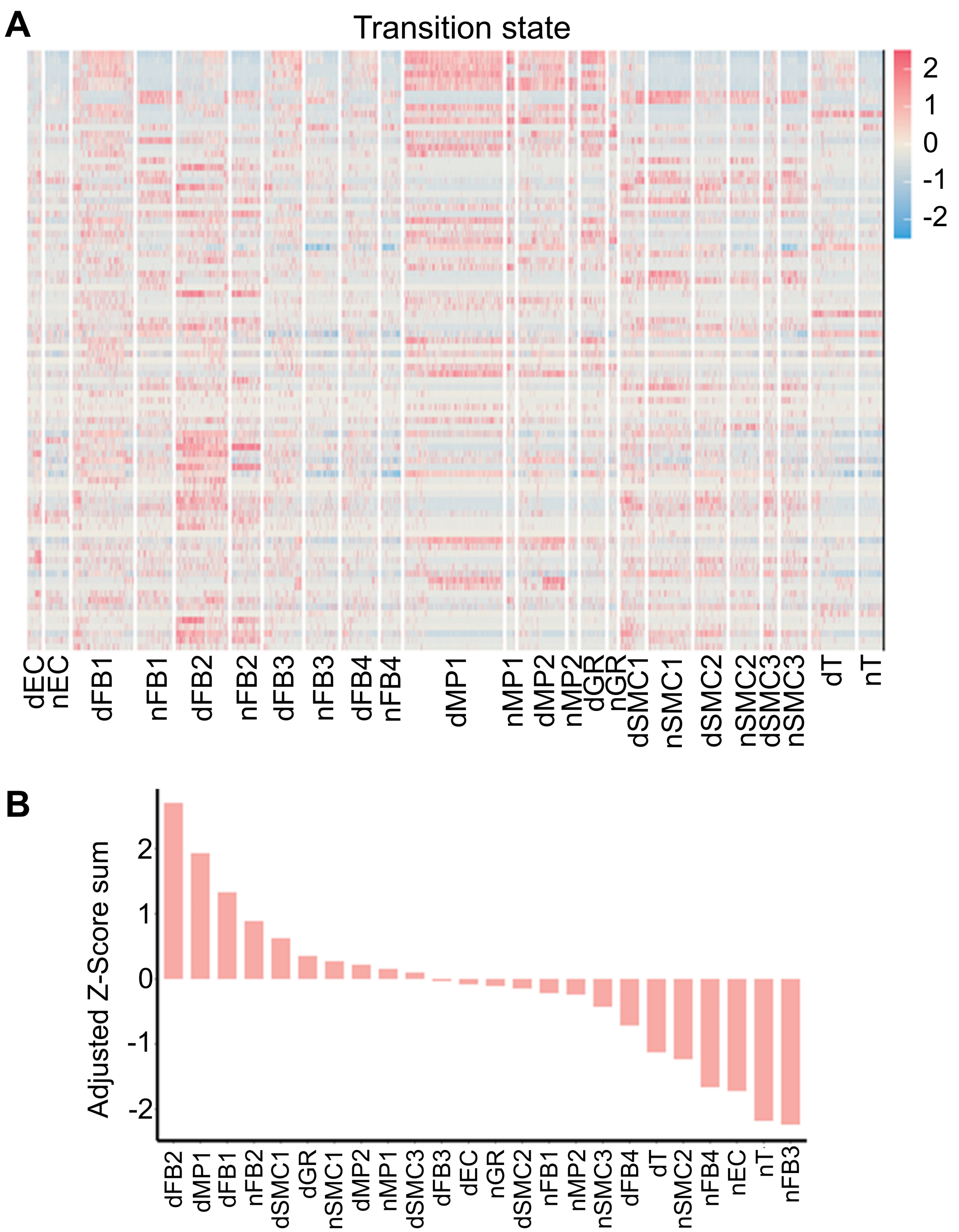

Supplemental figure 8. Quantification of putative cell-cell interactions in AD versus Non-AD. (A) Heatmap to show potential pairs between ligands (Supplemental figure 6C) expressed by indicated cell clusters and signaling pathways in transition state SMCs in Monocle analysis. **(B)** Sum of all matched pairs in (A).

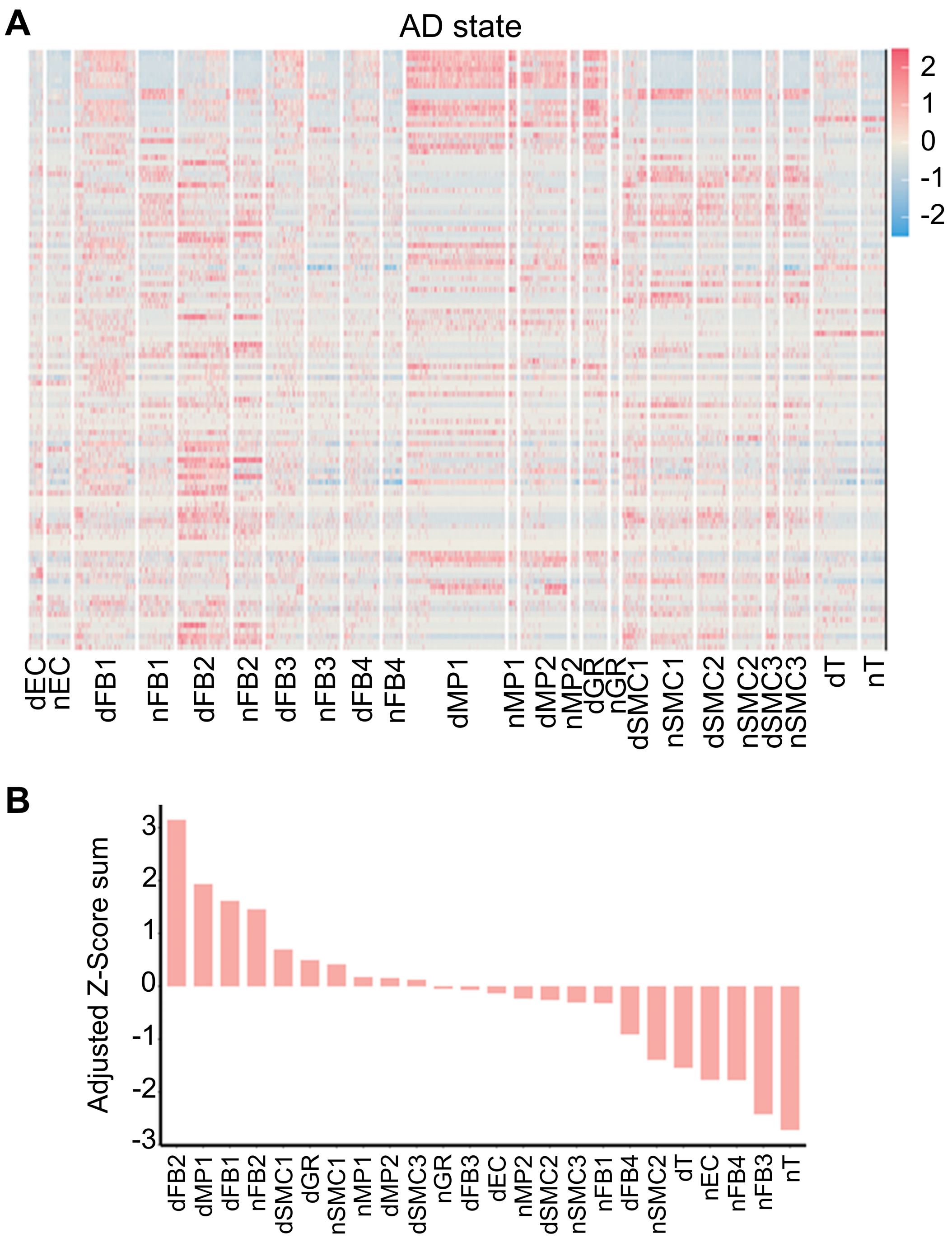

Supplemental figure 9. Quantification of putative cell-cell interactions in AD versus Non-AD. (A) Heatmap to show potential pairs between ligands (Supplemental figure 6C) expressed by indicated cell clusters and signaling pathways in AD state SMCs in Monocle analysis. **(B)** Sum of all matched pairs in (A).

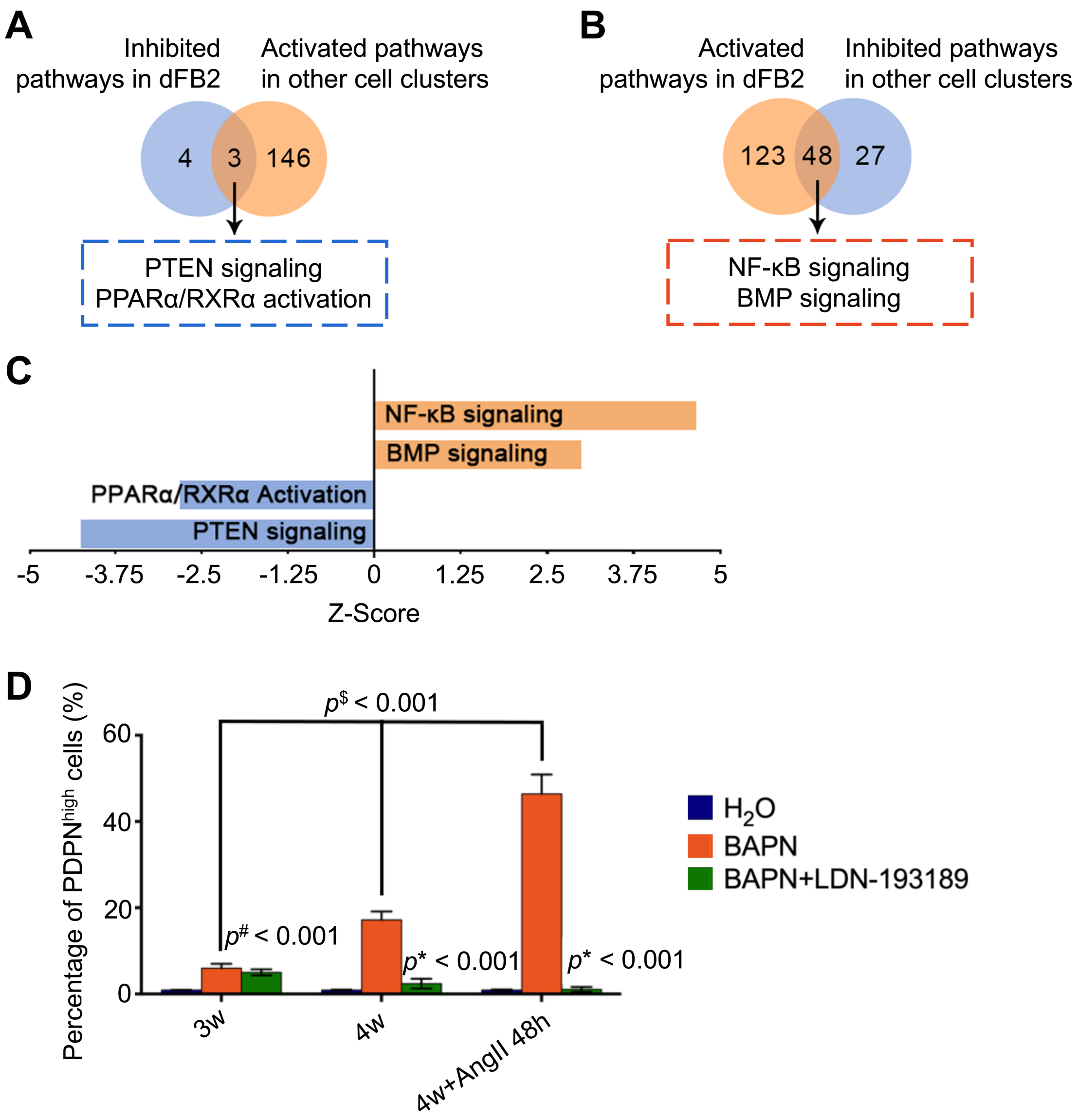

Supplemental figure 10. Targeting BMP signaling suppresses onset of AD. (A) Venn diagram to show the IPA pathways inhibited in dFB2 but activated in other cell clusters. **(B)** Venn diagram to show the IPA pathways activated in dFB2 but inhibited in other cell clusters. **(C)** *z*-score of indicated IPA canonical pathways in dFB2. A *z*-score < -2 indicates pathway inhibition; whereas a *z*-score > 2 indicates pathway activation of a pathway. **(D)** Quantification of PDPN^{high} cells in Figure 8H. n = 3. $p^{\$} < 0.001$, BAPN group versus H_2O group; $p\# < 0.001$,

BAPN+LDN-193189 group versus H_2O group; $p^* < 0.001$, BAPN+LDN-193189 group versus BAPN group (One-way ANOVA).

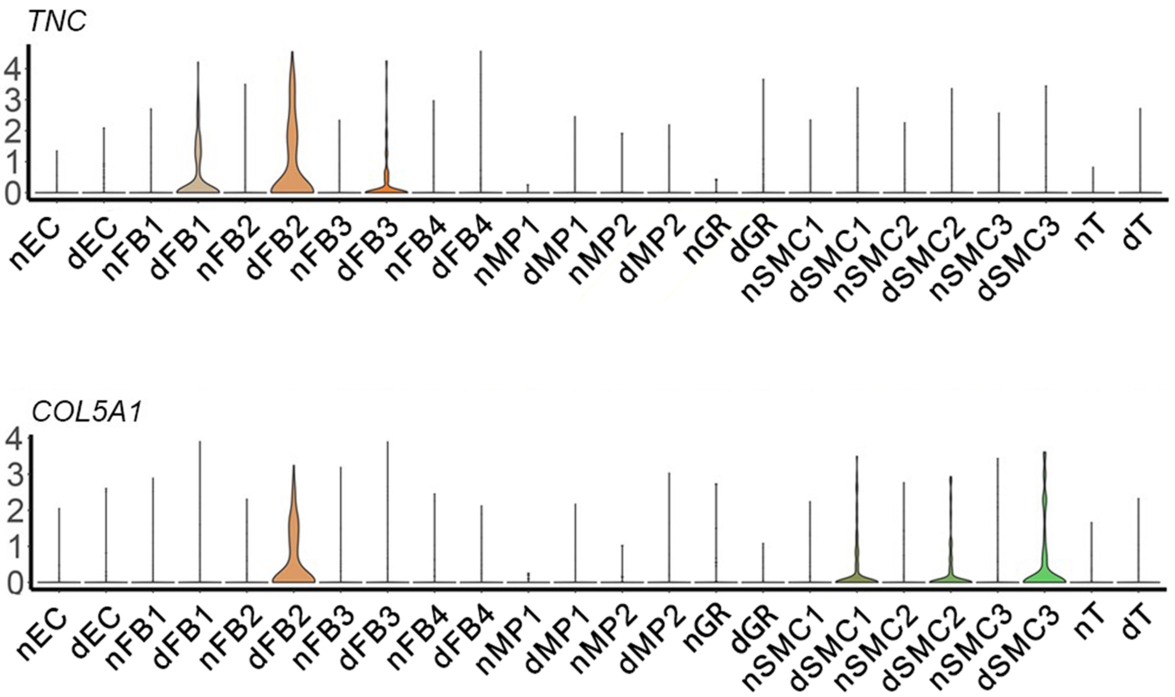

Supplemental figure 11. Expression levels of dFB2 specific ligands. (A, B) Expression of *TNC* (A) and *COL5A1* (B) in indicated cell clusters.

	Control (n = 30)	AD (n = 80)	*P*-value
Male, n (%)	23 (76.7%)	69 (86%)	NS
Age, Yr	34.9 ± 8.9	54.0 ± 13.0	< 0.001
Hypertension, n (%)	2 (6.7%)	65 (81.0%)	< 0.001
Smoking, n (%)	16 (53.3%)	37 (46.3%)	NS
Alcohol, n (%)	11 (36.7%)	34 (42.5%)	NS
DM, n (%)	3 (10%)	8 (10%)	NS
HR, bpm	76.3 ± 7.8	77.8 ± 12.7	NS
WBC, x 10^9/L	7.5 ± 3.8	8.8 ± 3.4	< 0.05
HGB, g/L	121.9 ± 21.0	127.1 ± 23.1	NS
FPG, mmol/L	5.2 ± 1.4	6.1 ± 1.8	< 0.05
CRP, mg/L	6.9 ± 1.3	63.7 ± 93.8	< 0.001

Data are presented as mean ± standard deviation. DM: diabetes mellitus; HR: heart rate; WBC: white blood cell; HGB: hemoglobin; FPG: fast plasma glucose; CRP: C-Reactive protein; NS: not significant

Supplemental figure 12. Clinical characteristics in patients who provide blood samples.
